# Analysis and Design of a Double-Chamber Centrifugal-Gyratory Mill for Fine and Ultrafine Comminution

**DOI:** 10.3390/ma19194036

**Published:** 2026-09-22

**Authors:** Margarita Aldanova, Bakhyt Absadykov, Erlan Askarov, Anna Kawałek, Alibek Ibraim, Assylbek Kassenov, Gulsunoy Abdukarimova, Tursunov Zakir

**Affiliations:** 1 Department of Mechanical Engineering, Satbayev University, Almaty 050013, Kazakhstan; aldanova_margari@inbox.ru (M.A.); ibraimaiibek@gmail.com (A.I.); 2 Department of Transport Engineering and Technologies, International Transport and Humanitarian University, Almaty 050000, Kazakhstan; erlan57@mail.ru; 3 Faculty of Production Engineering and Materials Technology, Czestochowa University of Technology, 42-201 Czestochowa, Poland; anna.kawalek@pcz.pl; 4 Department of Transport and Mechanical Engineering, Kazakh National University of Water Management and Irrigation, Taraz 080000, Kazakhstan; a.kassenov@kaznuvhi.edu.kz; 5 Faculty of Automotive Transport Engineering, Tashkent State Transport University, Tashkent 100167, Uzbekistan; gulsunoy_o@tstu.uz; 6 Faculty of Management of Transport Systems, Tashkent State Transport University, Tashkent 100167, Uzbekistan; tursunov@tstu.uz

**Keywords:** centrifugal gyratory mill, fine and ultrafine grinding, grinding chamber, grinding media, energy efficiency, operating parameters, raw minerals

## Abstract

This study presents the analysis, design, and experimental–numerical evaluation of a double-chamber centrifugal-gyratory mill developed for the fine and ultrafine grinding of mineral raw materials. The operating principle is based on plane-parallel circular motion of two cylindrical grinding chambers generated by a crank mechanism. This motion produces an intensified centrifugal field and promotes simultaneous impact, compression, abrasion, and interparticle breakage within the grinding zone. An analytical model was developed to evaluate the influence of crank radius, rotational speed, chamber geometry, and grinding-body mass on the forces governing material destruction. At a crank radius of 15 mm and a rotational speed of 500 min^−1^, the calculated centrifugal force acting on a 7 kg grinding body reached approximately 288 N, which is 4.2 times greater than its gravitational force. This confirms that the investigated grinding process is controlled predominantly by dynamically generated centrifugal loading rather than by gravity alone. Physical grinding tests demonstrated the ability of the mill to fracture hard mineral samples under intensive operating conditions, confirming the practical effectiveness of the combined impact–compression mechanism. Structural performance was assessed using global and local finite element models developed in Autodesk Fusion. The complete double-chamber assembly, subjected to three applied moments of 900 N·m, exhibited a maximum von Mises stress of 22.915 MPa, a maximum displacement of 0.050 mm, and a minimum safety factor of 9.033. The local shaft–chamber–support model, analysed under a torque of 500 N·m, produced a maximum stress of 65.837 MPa, a displacement of 0.740 mm, and a minimum safety factor of 3.144. In both numerical models, the maximum stresses remained substantially below the assigned steel yield strength of 207 MPa. The combined analytical, experimental, and numerical results demonstrate that the proposed mill generates sufficient dynamic loading for hard-rock breakage while maintaining adequate structural strength, stiffness, and operational safety under high-torque conditions.

## 1. Introduction

Crushing and grinding involve different mechanisms of particle-size reduction. Crushing is predominantly achieved through compressive forces, whereas impact and abrasion generally play secondary roles. In grinding processes, abrasion and impact become considerably more important. Grinding can be performed under either dry or wet conditions, depending on the material properties and technological requirements. Grinding media are commonly spherical or cylindrical bodies that promote particle breakage through repeated impact, compression, and abrasive interactions with the processed material. In continuous grinding systems, the feed material enters the mill at one end, while the ground product is discharged at the opposite end. The selection of an appropriate size-reduction strategy and grinding equipment is strongly influenced by the energy required to achieve the target particle size. This energy demand, in turn, depends on the initial particle size, material hardness and other mechanical properties, as well as the required characteristics of the final product.

Historically, fine grinding was considered undesirable in the mining industry because of the limitations associated with conventional grinding equipment, particularly tumbling mills. For hard minerals, achieving the required degree of fineness often required prolonged grinding times, resulting in limited throughput and increased energy consumption [1]. Depending on the target product size, comminution can involve several successive grinding stages. Regrinding is commonly applied to produce particles smaller than approximately 75 µm and, in some cases, down to approximately 30 µm. Fine grinding generally extends the particle-size range further, while ultrafine grinding is employed when particles below approximately 10 µm are required. At even smaller scales, mechanical grinding can be combined with other processing or synthesis techniques to obtain materials with particle dimensions approaching the nanometer range [2,3].

Energy consumption and the wear of steel components, including grinding media and mill liners, contribute substantially to the overall cost of comminution. Among mineral processing operations, comminution is recognized as one of the most energy-intensive stages. Ore grinding, in particular, accounts for a considerable proportion of the total energy consumed in mineral processing [4]. As the required product size decreases, the specific energy demand generally increases, making energy efficiency increasingly important in fine and ultrafine grinding applications.

The growing demand for mineral resources, cement, and finely ground industrial materials requires increasingly intensive comminution operations. Producing finer products generally involves additional energy expenditure [5]. Previous studies have reported that comminution represents a substantial share of the total energy consumption of mining operations [6]. Therefore, the development of more energy-efficient grinding technologies remains an important engineering challenge. The selection of a sui Standard mill depends on the required degree of size reduction, the properties of the feed material, the desired product-size distribution, and the associated energy consumption. Consequently, minimizing specific energy consumption while maintaining the required grinding performance is one of the principal objectives in the development of modern comminution equipment.

Modernization of existing machines and the development of new grinding equipment aimed at reducing energy consumption and increasing productivity are therefore among the major challenges addressed by equipment manufacturers and research institutions. Reducing grinding costs directly affects the overall production cost and, consequently, the economic competitiveness of the final product.

In conventional crushers and mills, rock particles are primarily broken by compression, abrasion, and impact because these loading mechanisms can be implemented relatively easily in mechanical equipment. However, rocks may exhibit considerable resistance to compression and abrasion. Alternative modes of loading, including bending and tensile stresses, may under certain conditions promote fracture at lower energy expenditure. This indicates that improving the way mechanical energy is transferred to particles is an important direction for increasing comminution efficiency.

In this context, grinding systems characterized by increased velocities of the working components and intensified dynamic loading are of particular interest. Centrifugal-gyratory mills represent one such approach. Their operating principle makes it possible to generate an intensified force field within the grinding chamber, thereby increasing the frequency and intensity of interactions among the grinding bodies, material particles, and chamber walls. As a result, these mills have the potential to provide higher grinding intensity and productivity than conventional grinding systems of comparable dimensions [7,8]. These characteristics provide the basis for further investigation of centrifugal-gyratory mills as a potential solution for energy-efficient fine and ultrafine grinding applications.

Comminution is a fundamental operation in mineral processing, construction-material production, metallurgy, ceramics, and chemical engineering. The particle-size distribution obtained during grinding directly affects mineral liberation, specific surface area, reaction kinetics, mixing behaviour, downstream separation efficiency, and the quality of the final product. Despite its technological importance, grinding remains one of the most energy-intensive stages of raw-material processing. A considerable proportion of the mechanical energy supplied to conventional mills is dissipated through non-productive movement of the grinding media, friction, heat generation, vibration, noise, liner deformation, and wear. These losses become increasingly significant as the required product size decreases because fine and ultrafine comminution demand a greater number of repeated loading events and more intensive energy transfer to progressively smaller material volumes [9,10].

Conventional drum and ball mills remain widely used because of their relatively simple construction, operational reliability, and ability to process large quantities of mineral feed. Their grinding action is mainly governed by gravitational lifting, cascading, and falling of the grinding media. Consequently, the intensity of particle breakage depends strongly on drum diameter, rotational speed, chamber filling degree, media size, and the height from which the grinding bodies fall. At insufficient rotational speed, the media may roll or slide without generating adequate impact energy. Conversely, excessive speed may cause the grinding bodies to remain attached to the drum wall, resulting in an undesirable centrifuging regime and a reduction in effective collisions. These limitations restrict the flexibility of conventional mills, particularly when processing hard mineral materials or producing fine and ultrafine fractions [11].

To overcome these limitations, alternative grinding systems have been developed in which additional dynamic loading is imposed on the grinding chamber or grinding media. Vibratory, planetary, stirred-media, and centrifugal mills generate higher accelerations and more frequent particle–media interactions than gravity-dominated drum systems. Their principal advantage is the possibility of combining several particle-breakage mechanisms, including impact, compression, abrasion, shear, and interparticle fracture. However, dynamically activated mills can also be characterised by complex drive systems, substantial bearing loads, high vibration levels, limited chamber capacity, and demanding balancing requirements. Their successful application therefore depends not only on grinding intensity but also on the structural strength, stiffness, kinematic stability, and service life of the complete machine [12].

The centrifugal-gyratory mill investigated in the present study applies a different principle of mechanical activation. The machine contains two horizontal cylindrical grinding chambers mounted in parallel on a common steel supporting structure. Unlike conventional rotating drums, the chambers do not rotate continuously about their own longitudinal axes. Instead, both cylindrical bodies perform synchronous plane-parallel circular motion generated by a crank mechanism. Each point of a chamber follows a circular trajectory whose radius is determined by the crank eccentricity, while the chamber axes remain parallel throughout the operating cycle.

A distinctive feature of the developed design is the use of a single 3 kW electric motor to drive both grinding chambers. Mechanical power is transmitted from the motor to the primary shaft and crank assembly. The first chamber receives motion directly from the driven crankshaft, whereas the second chamber is mechanically coupled to the first through a parallelogram-type crank linkage. The connecting plates and crank elements form a closed kinematic contour that maintains the relative orientation of the chambers and ensures synchronous movement. This single-motor configuration eliminates the need for independent speed control of two drives and prevents phase drift between the grinding chambers. As a result, both chambers operate at the same excitation frequency and with a mechanically defined phase relationship.

The principal machine geometry is based on the assembly design of the CM-5 centrifugal mill. The mill has an overall length of approximately 1615 mm and a width of about 1000 mm. The external diameter of each cylindrical chamber is approximately 420–426 mm, and the nominal shaft speed is 750 min^−1^. Approximately 50 kg of raw material can be loaded into each chamber, providing a total nominal feed capacity of about 100 kg per operating cycle. The two chambers, crankshafts, connecting plates, bearings, and support elements are installed on a common welded frame that transfers the dynamic reactions to the foundation [13].

The imposed plane-parallel motion generates a periodically changing inertial field inside each chamber. The grinding bodies and mineral particles are therefore subjected to forces substantially different from those occurring in conventional gravity-driven mills. The chamber motion causes the grinding bodies to interact repeatedly with the processed material and chamber walls. Depending on their instantaneous angular position, the grinding bodies may accelerate along the internal surface, increase the normal compression of particles against the wall, or resist the relative movement of the material bed. The resulting grinding environment combines repeated impacts, wall-contact compression, abrasion, friction, shear, and interparticle breakage [14,15].

The performance of the mill is governed by a coupled set of geometrical, kinematic, and material parameters. These include chamber diameter and length, crank eccentricity, shaft speed, number, dimensions, and mass of the grinding bodies, feed loading, chamber filling degree, initial particle size, material hardness, friction conditions, and resistance generated by the processed mineral bed. The internal circulation speed of the grinding bodies is generally lower than the shaft speed because their motion is opposed by friction, particle resistance, impacts, and interaction with neighbouring grinding bodies. Nevertheless, the repeated circulation of the media through active impact and compression zones provides the mechanical basis for progressive particle-size reduction [16].

The single-motor parallelogram arrangement also influences the structural behaviour of the machine. Because the two chambers are mechanically linked, the motion and reaction forces of one chamber are transferred through the connecting plates and crank elements to the second chamber and to the common frame. This arrangement improves kinematic synchronisation but introduces cyclic loading into the shafts, bearings, chamber supports, linkage plates, and welded frame members. The principal structural elements are therefore subjected to combined torque, bending, eccentric inertia forces, contact reactions, and impact-induced loading [17].

Increased grinding intensity can produce substantial mechanical loads. For this reason, the performance of the mill cannot be evaluated only by its ability to fracture mineral material. The structural system must also preserve shaft alignment, chamber positioning, crank geometry, and support rigidity during continuous operation. Excessive displacement may lead to bearing misalignment, non-uniform load distribution, contact between adjacent components, accelerated wear, vibration growth, and fatigue damage. Structural verification is therefore an essential part of the development of centrifugal-gyratory grinding equipment [18].

Previous investigations of centrifugal and gyratory mills have mainly focused on their operating principles, kinematic schemes, grinding mechanisms, or potential energy advantages. The relationship between a single-motor mechanically synchronised double-chamber design, experimental hard-rock fracture, and the stress–strain response of the complete structure has received less systematic attention. In particular, limited information is available on machines in which two parallel grinding chambers are connected through a parallelogram crank mechanism and operate as one kinematically coupled system.

The present study addresses this research gap through an integrated analytical, experimental, and numerical investigation of a double-chamber centrifugal-gyratory mill. Physical grinding tests are used to evaluate the ability of the proposed mechanism to fracture hard mineral feed under intensive operating conditions. Analytical calculations are applied to describe the main force relationships and the motion of the grinding bodies. In parallel, finite element simulations are used to assess two principal structural subsystems: the complete double-chamber cylindrical assembly and the critical crusher-shaft and chamber-support mechanism. The numerical analyses evaluate equivalent stress, displacement, contact loading, reaction forces, and safety factors under amplified static loading conditions.

The scientific novelty of the work lies in the combined evaluation of centrifugal-gyratory grinding mechanics, single-motor chamber synchronisation, experimental hard-rock breakage, and multiscale structural behaviour. The study examines not only the force field responsible for particle destruction but also the capacity of the crank-linked chamber system to transmit mechanical power without yielding or excessive deformation. The main objective is to establish whether the proposed single-motor, double-chamber design can generate sufficiently intensive loading for fine and ultrafine grinding while preserving adequate structural strength, stiffness, kinematic synchronisation, and operational stability. The obtained results provide an engineering basis for assessing the centrifugal-gyratory mill as a compact and mechanically robust alternative to conventional gravity-dominated grinding equipment [19,20,21,22].

## 2. Materials and Methods

### 2.1. Mill Design and Operating Principle

Recent developments in Structural Health Monitoring (SHM) demonstrate the increasing potential of data-driven methods for evaluating the condition and reliability of mechanical structures. Recent comprehensive studies have highlighted the integration of machine-learning and deep-learning techniques into SHM for automated damage detection, feature extraction, and structural-condition assessment [23]. Frequency Response Function (FRF) data combined with artificial neural networks and optimization algorithms have also demonstrated considerable potential for vibration-based damage identification [24]. More recently, hybrid machine-learning approaches based on vibration and modal data have been proposed for structural damage prediction and recovery assessment [25]. These developments are particularly relevant to rotating mechanical systems such as centrifugal-gyratory mills, in which crank mechanisms, shafts, bearing assemblies, and chamber supports are subjected to repeated cyclic and dynamic loading. Although data-driven SHM methods are beyond the scope of the present study, vibration-based monitoring combined with machine-learning techniques represents a promising direction for future assessment of the structural condition, early damage detection, and long-term reliability of the proposed mill [23,24,25].

The investigated machine is a double-chamber centrifugal-gyratory mill (Kazakh National Research Technical University named after K.I. Satpayev, Almaty, Kazakhstan) developed for the fine and ultrafine grinding of mineral raw materials. Its design combines two horizontally arranged cylindrical grinding chambers with a crank-driven support system, bearing units, connecting shafts, side brackets, a welded steel frame, and an electric drive with a reduction transmission. The double-chamber arrangement was selected to increase the working volume of the mill while maintaining a compact overall structure. It also allows two grinding chambers to operate simultaneously within a common mechanical system, thereby increasing potential throughput and distributing the working load between parallel grinding units. The general arrangement of the mill and the relative positions of the chambers, crankshafts, supports, and drive-side elements are shown in the three-dimensional model and kinematic scheme. Figure 1 shows the front view of two chambers.

The operating principle differs fundamentally from that of conventional rotating drum and ball mills. In a traditional drum mill, the cylindrical shell rotates about its longitudinal axis, lifting the grinding media until gravitational forces cause them to cascade or fall onto the processed material. In the proposed centrifugal-gyratory mill, the two horizontal grinding chambers do not rotate about their own axes. Instead, they perform synchronous plane-parallel circular motion generated by a single motor-driven crank system. The first chamber receives motion from the primary crankshaft, while the second chamber is mechanically coupled through a parallelogram-type linkage formed by the connecting plates and crank elements. This arrangement maintains the parallel orientation of both chamber axes and ensures that their centres follow coordinated circular trajectories with a radius determined by the crank eccentricity. Consequently, every point of each chamber undergoes periodic circular translation in a plane perpendicular to the chamber axis. The resulting motion generates a continuously varying inertial force field inside both chambers, increasing the relative movement and contact frequency between the grinding bodies, mineral particles, and chamber walls. This intensified interaction promotes combined impact, compression, abrasion, shear, and interparticle breakage, thereby providing more active energy transfer to the processed material than gravity-dominated grinding systems [26]. Figure 2 illustrates a side-view blueprint of the double-chamber centrifugal-gyratory mill, showing the motor, reducer shaft, grinding chambers, supporting structure, and drive system.

The power-transmission system consists of a single 3 kW electric motor, a primary drive shaft, eccentric crank components, chamber-support shafts, and a parallelogram linkage connecting the two grinding chambers. The motor drives the primary shaft at a nominal speed of 750 min^−1^. The eccentric crank mechanism converts shaft rotation into plane-parallel circular motion, while the parallelogram linkage ensures synchronous motion and parallel alignment of both chambers.

The frame, supports, brackets, bearings, and chamber-support shafts form the main load-bearing structure. These components transfer motor torque, inertia forces, chamber reactions, and grinding-induced loads to the foundation while maintaining shaft alignment and chamber parallelism during operation. The principal power-flow sequence can therefore be expressed as:

The mill has an external chamber diameter of approximately 420–426 mm and an overall length of approximately 1615 mm. Each chamber has a nominal feed capacity of approximately 50 kg. For the analytical model, the effective internal working diameter was 140 mm. Four cylindrical grinding bodies, each 45 mm in diameter and approximately 7 kg in mass, were installed in each chamber.

The average circulation speed of the grinding bodies was approximately 170 min^−1^, compared with the shaft speed of 750 min^−1^. This difference results from wall friction, material resistance, impacts between neighbouring grinding bodies, and the continuously varying inertia force. The drive and chamber-synchronisation arrangement is shown in Figure 3.

$$\begin{array}{c} \mathrm{Electric}\;\mathrm{motor}\to \mathrm{primary}\;\mathrm{drive}\;\mathrm{shaft}\to \mathrm{eccentric}\;\mathrm{crank}\;\mathrm{mechanism}\to \mathrm{Chamber}\;1\\ \to \mathrm{parallelogram}\;\mathrm{linkage}\to \mathrm{Chamber}\;2. \end{array}$$

Figure 3 presents the main functional configuration of the double-chamber centrifugal-gyratory mill and illustrates the relationship between the drive system and the two grinding chambers. The system consists of a control panel, a 3 kW electric motor, a reduction unit, the main drive shaft, and two cylindrical grinding chambers (Chambers 1 and 2) arranged within a common mechanical system. The electric motor transmits torque through the reduction unit to the main drive shaft, while the crank mechanism converts the rotational motion of the shaft into plane-parallel circular motion of the grinding chambers. The two-chamber arrangement allows the chambers to operate simultaneously and is intended to provide a more symmetric distribution of the working load during normal operation. The principal overall dimensions of the mill are approximately 1615 mm in length and 420–426 mm in external chamber diameter. For the analytical model, the effective internal working diameter of each chamber was taken as 140 mm.

During operation, the feed material and four cylindrical grinding bodies are loaded into each grinding chamber. The chamber covers are then secured, and the single 3 kW electric motor accelerates the primary drive shaft and crank mechanism to the selected operating speed. Motion is transmitted from the driven chamber to the second chamber through two parallel connecting plates and associated crank elements forming a parallelogram linkage. This mechanical connection ensures that both chambers move synchronously and that their longitudinal axes remain parallel throughout the operating cycle.

The nominal shaft speed of the full-scale mill is 750 min^−1^, while approximately 50 kg of raw material can be loaded into each chamber. At a crank eccentricity of 15 mm, the chamber acceleration is determined as

(1) $$a_{c}=r\omega_{c}^{2},\;\;$$

where *r* is the crank eccentricity and $\omega_{c}$ is the angular velocity of the crankshaft. At 750 min^−1^, the angular velocity is approximately 78.54 rad/s, producing a chamber acceleration of approximately 92.5 m/s^2^. This value corresponds to approximately 9.4 times gravitational acceleration. Consequently, the motion of the grinding bodies is governed mainly by the periodically changing inertia field generated by the chamber rather than by gravity alone.

The imposed circular translation presses the grinding bodies against the internal chamber surface while simultaneously driving them along the chamber wall. Their motion creates a circulating and continuously redistributed material bed. Coarse particles are repeatedly captured between a grinding body and the chamber wall, between neighbouring grinding bodies, and within compressed layers of mineral feed. Because both chambers are mechanically connected to the same crank system, they operate at an identical excitation frequency and maintain a fixed kinematic relationship without requiring separate motor-speed control [27,28,29,30].

Particle breakage occurs through several complementary mechanisms. Impact loading is generated when the grinding bodies accelerate relative to the mineral particles and collide with coarse fragments, neighbouring bodies, or concentrated regions of the material bed. Compression occurs when particles become trapped in narrowing contact zones between the cylindrical grinding bodies and the internal chamber surface. Abrasion and shear develop as the grinding bodies slide or roll along the wall while applying normal pressure to the processed material. Interparticle breakage also occurs because particles of different sizes and masses move at different velocities and repeatedly interact within the compressed material layer.

The relative importance of these mechanisms changes as grinding proceeds. Coarse feed particles are initially fractured primarily by impact and concentrated compression. As the average particle size decreases, abrasion, shear, repeated low-energy compression, and particle-to-particle interaction become increasingly important. The combined action of these mechanisms provides the physical basis for progressive fine and ultrafine particle-size reduction [31].

The angular position of each grinding body affects its instantaneous contribution to the comminution process. Because the direction of the chamber-induced inertia force changes continuously during one crank revolution, its radial and tangential components relative to each grinding body also vary with time. At certain angular positions, the tangential component accelerates a body along the chamber wall. At other positions, the radial component increases the normal contact force between the grinding body, material bed, and chamber surface. During another portion of the cycle, the inertia force may partially oppose the relative movement of the body.

The four grinding bodies in each chamber therefore form a coupled mechanical system. At any instant, one or more bodies may provide the principal impact or driving action, whereas the remaining bodies compress, redistribute, or stabilise the material bed. This interaction explains why the average circulation speed of the grinding bodies is lower than the crankshaft speed, even though the bodies remain subjected to substantial cyclic inertia loading. Their relative motion is reduced by wall friction, mineral-bed resistance, impacts, and interaction with neighbouring grinding bodies [32].

The use of two mechanically linked chambers provides both operational and structural advantages. Processing capacity is increased without requiring a single chamber of substantially greater diameter. At the same time, both chambers are driven by one motor and one primary crank system, reducing the complexity associated with independent drives and eliminating relative speed and phase drift between the chambers. The parallelogram linkage distributes motion through the connecting plates and maintains the required parallel orientation of the chamber axes.

However, the mechanically coupled arrangement also transfers cyclic forces between the two chambers and into the common supporting structure. The frame, main supports, chamber brackets, crankshafts, bearings, and connecting plates must therefore withstand combined motor torque, eccentric inertia forces, grinding reactions, and impact-induced loading. The machine structure must also preserve shaft alignment and chamber clearance under continuous cyclic operation [33].

The global and local finite element models showed that the principal structural elements remained below the assigned steel yield strength under the investigated amplified static loading conditions. In the complete-machine model, the maximum equivalent stress was 22.915 MPa, the maximum displacement was 0.050 mm, and the minimum safety factor was 9.033. In the local shaft–chamber-support model, the corresponding maximum stress was 65.837 MPa, the maximum displacement was 0.740 mm, and the minimum safety factor was 3.144. These results indicate adequate static structural capacity, although fatigue and transient dynamic effects require separate evaluation because the mill operates under repeated cyclic excitation [34,35].

### 2.2. Experimental Grinding Procedure

Experimental grinding tests were conducted using the manufactured double-chamber centrifugal-gyratory mill prototype to evaluate its ability to fracture mechanically resistant mineral material and to assess the operational stability of the coupled chamber system.

Before each test, the grinding chambers, grinding bodies, crank mechanism, bearings, supports, and connecting elements were visually inspected. The feed material was prepared and weighed, and four cylindrical grinding bodies were installed in the loaded chamber. The chamber cover and retaining elements were then secured.

The mill was driven by a single 3 kW electric motor. The experimental procedure included control of the chamber loading, grinding-body arrangement, shaft speed, processing duration, and post-grinding evaluation of the material [36]. Figure 4 presents the three-dimensional model of the double-chamber centrifugal-gyratory mill developed in Autodesk Fusion 360.

Experimental grinding tests were conducted using the manufactured double-chamber centrifugal-gyratory mill prototype. During the investigated test, 50 kg of hard mineral feed was loaded into one grinding chamber, while the second chamber was not loaded with raw material. The purpose of this configuration was to evaluate the capability of the single driven chamber to fracture mechanically resistant mineral material and to assess the structural behaviour of the mill under an intentionally asymmetric operating load. The original CM5 assembly drawing specifies a recommended feed quantity of approximately 50 kg for each drum; however, only one chamber was filled during the present experimental regime. 

The feed consisted of hard mineral fragments selected to represent mechanically resistant raw material. Before testing, the material was cleaned of loose contamination, dried, and visually inspected to remove metallic objects and other foreign inclusions that could damage the chamber or influence the grinding results. The initial feed mass was measured as

(2) $$m_{f}=50\;\mathrm{kg}$$

Representative samples of the initial material were retained for comparison with the ground product. The mineral composition, density, hardness, compressive strength, moisture content, and initial particle-size distribution should be reported together with the experimental results [17,18].

Four cylindrical steel grinding bodies were installed in the loaded chamber. Each grinding body had a diameter of 45 mm and a mass of approximately 7 kg. The total mass of the grinding bodies was therefore

(3) $$m_{b}=4\times 7=28\;\mathrm{kg}$$

The total internal moving mass in the loaded chamber, excluding the mass of the chamber itself, was

(4) $$m_{int}=m_{f}+m_{b}$$

$$m_{int}=50+28=78\;\mathrm{kg}$$

The selected filling condition provided sufficient mineral material for repeated particle–body, particle–wall, and particle–particle interactions while retaining free space for the circulation of the grinding bodies. Excessive filling was avoided because a densely compacted material bed could restrict grinding-body movement, decrease impact velocity, and increase the risk of local jamming.

The chamber and primary shaft were manufactured from 40X chromium-alloy structural steel. This material was selected because these components are exposed to cyclic bending, torsion, contact pressure, impact loading, and abrasive wear. The actual mechanical properties used in the structural calculations should correspond to the material certificate and heat-treatment condition of the manufactured components.

After loading, the chamber cover and all retaining elements were secured. The crankshafts, bearings, chamber supports, parallelogram connecting plates, and mechanical joints were inspected before starting the mill. A single 3 kW electric motor actuated the primary drive shaft. The crank mechanism converted shaft rotation into plane-parallel circular displacement of the chamber, while the parallelogram linkage maintained the parallel orientation of the two cylindrical chambers.

The nominal operating speed was

(5) $$n_{c}=750\;\min^{-1}$$

and the crank eccentricity was

e=15 mm=0.015 m

During operation, the mill was continuously monitored for abnormal vibration, unstable chamber motion, excessive bearing temperature, loosening of mechanical connections, unusual impact noise, and possible interference between moving components.

Particle breakage occurred through a combination of impact, compression, abrasion, shear, and interparticle fracture. Coarse mineral fragments were initially broken by impacts from the cylindrical grinding bodies. Particles trapped between the grinding bodies and the chamber wall were subjected to concentrated compression. Sliding and rolling of the grinding bodies generated abrasion and shear, while continuous redistribution of the feed produced repeated particle-to-particle interaction [37].

At the end of the selected grinding period, the electric motor was stopped and the chambers were allowed to come to a complete rest. The processed material was collected from the loaded chamber and compared with the initial feed using photographs, mass measurements, and particle-size evaluation. The chamber surface, 40X steel shaft, grinding bodies, bearings, brackets, and connecting plates were inspected for visible deformation, cracking, displacement, abnormal wear, or loosening [38,39,40].

### 2.3. Analytical Load Model

The analytical model was developed to estimate the principal inertia and gravitational loads generated by the 50 kg feed and four grinding bodies in one chamber. The calculated values represent ideal harmonic load amplitudes associated with the plane-parallel motion of the chamber. They do not include short-duration impact peaks, the mass of the chamber itself, or dynamic amplification caused by structural resonance.

The crank angular velocity is

(6) $$\omega_{c}=\frac{2\pi n_{c}}{60}$$

For a shaft speed of 750 min^−1^,

(7) $$\omega_{c}=\frac{2\pi \times 750}{60}=78.54\;\mathrm{rad}/s$$

The chamber-centre acceleration amplitude is

(8) $$a_{c}=e\omega_{c}^{2}$$

For a crank eccentricity of 0.015 m,

$$a_{c}=0.015\times 78.54^{2}=92.53\;{m/s}^{2}$$

The acceleration ratio relative to gravity is

(9) $$\Gamma =\frac{a_{c}}{g}\Gamma =\frac{92.53}{9.81}=9.43$$

Thus, the chamber-generated acceleration is approximately 9.43 times gravitational acceleration.

Load generated by the 50 kg feed.

The ideal inertia-force amplitude associated with the feed mass is

(10) $$F_{f}=m_{f}a_{c}$$

Therefore,

$$F_{f}=50\times 92.53=4626.5\;N$$

The gravitational force of the feed is

(11) $$G_{f}=m_{f}gG_{f}=50\times 9.81=490.5\;N$$

Therefore, the chamber motion generates an ideal inertia force on the feed approximately 9.43 times greater than its weight.

Load generated by one grinding body.

For one grinding body with a mass of 7 kg,

(12) $$\begin{array}{c} F_{b,1}=m_{b,1}a_{c}\\ F_{b,1}=7\times 92.53=647.7\;N \end{array}$$

Its gravitational force is

(13) $$G_{b,1}=7\times 9.81=68.7\;N$$

Total load generated by four grinding bodies.

For four grinding bodies,

(14) $$\begin{array}{c} F_{b,total}=4F_{b,1}\\ F_{b,total}=4\times 647.7=2590.8\;N \end{array}$$

Their total gravitational force is

(15) $$G_{b,total}=28\times 9.81=274.7\;N$$

Combined internal load in the loaded chamber.

The total internal mass is

(16) $$m_{int}=78\;\mathrm{kg}$$

The combined inertia-force amplitude is

(17) $$\begin{array}{c} F_{int}=m_{int}a_{c}\\ F_{int}=78\times 92.53=7217.3\;N \end{array}$$

The total gravitational force is

(18) $$G_{int}=78\times 9.81=765.2\;N$$

Depending on the crank position, gravity may act in the same direction as or opposite to the chamber-induced inertia force. The approximate maximum combined load is therefore

(19) $$\begin{array}{c} F_{max}=F_{int}+G_{int}\\ F_{max}=7217.3+765.2=7982.5\;N \end{array}$$

The approximate minimum resultant load in the opposite orientation is

(20) $$\begin{array}{c} F_{min}=F_{int}-G_{int}\\ F_{min}=7217.3-765.2=6452.1\;N \end{array}$$

Accordingly, the loaded chamber is subjected to a rotating internal force with an ideal amplitude of approximately 7.22 kN, while the combined instantaneous force may vary approximately between 6.45 and 7.98 kN, depending on the angular relationship between inertia and gravity. The calculated load components and their corresponding inertia and gravitational forces are summarized in Table 1.

These values exclude the inertia of the 40X steel chamber, covers, shafts, supports, and crank components. When the chamber moving mass $m_{ch}$ is known, its additional inertia-force amplitude should be calculated as

(21) $$F_{ch}=m_{ch}e\omega_{c}^{2}=92.53m_{ch}$$

Therefore, every additional kilogram of moving chamber and support mass produces approximately

(22) 92.53 N

of cyclic inertia-force amplitude at 750 min^−1^ and an eccentricity of 15 mm.

Because only one chamber contained 50 kg of feed, the system operated under an asymmetric load. The additional unbalanced inertia force caused solely by the feed was approximately 4.63 kN. This load was transferred through the 40X steel chamber, crankshaft, bearings, parallelogram connecting plates, main supports, and welded frame. The asymmetric condition should therefore be considered in the finite element model as a critical experimental load case.

The nominal torque available from the 3 kW motor at 750 min^−1^ is

(23) $$\begin{array}{c} T_{m}=\frac{9550P}{n}\\ T_{m}=\frac{9550\times 3}{750}=38.2\;N\backslash \mathrm{cdotpm} \end{array}$$

This is the continuous motor torque and should not be confused with the cyclic inertia forces calculated above. The actual shaft and crank reactions depend on the chamber mass, phase geometry, transmission efficiency, material movement, bearing resistance, and impact loading. Consequently, the calculated 7.22 kN represents the ideal internal inertia-load amplitude, while the design load should include an experimentally justified dynamic amplification coefficient. Figure 5 illustrates the kinematic scheme of the working mechanism [41].

The relative intensity of the imposed dynamic loading was evaluated using the dimensionless acceleration ratio

(24) $$\Gamma =\frac{a_{c}}{g}=\frac{F_{c}}{F_{g}}=\frac{r\omega_{c}^{2}}{g}$$

For the selected operating regime of

$$n_{c}=750\;\min^{-1},e=0.015\;m$$

the crank angular velocity is

(25) $$\omega_{c}=\frac{2\pi n_{c}}{60}=\frac{2\pi \times 750}{60}=78.54\;\mathrm{rad}/s$$

The corresponding chamber acceleration is

$$a_{c}=e\omega_{c}^{2}=0.015\times 78.54^{2}=92.53\;{m/s}^{2}$$

Therefore, the acceleration ratio is

(26) $$\Gamma =\frac{92.53}{9.81}\approx 9.43$$

The same result is obtained from the force ratio for one 7 kg grinding body:

(27) $$\begin{array}{c} F_{c}=m_{b}a_{c}=7\times 92.53=647.7\;N\\ F_{g}=m_{b}g=7\times 9.81=68.67\;N\\ \Gamma =\frac{647.7}{68.67}\approx 9.43 \end{array}$$

Because Γ>1, the movement of the grinding bodies is governed primarily by chamber-induced inertial loading rather than by gravity. This distinguishes the investigated centrifugal-gyratory mill from conventional drum mills, in which lifting and gravitational falling of the grinding media represent the dominant breakage mechanism. In the proposed mill, the periodically changing inertia force maintains intensive interaction between the grinding bodies, mineral particles, and chamber wall throughout a substantial part of the operating cycle [42].

The inertia force increases with the square of rotational speed:

(28) $$F_{c}\propto n_{c}^{2}$$

For example, increasing the crank speed from 500 to 750 min^−1^ gives

$$\frac{F_{c,750}}{F_{c,500}}={\left(\frac{750}{500}\right)}^{2}=2.25$$

Thus, a 50% increase in shaft speed produces a 125% increase in inertia-force amplitude. For one 7 kg grinding body, the force increases from approximately 287.9 N at 500 min^−1^ to approximately 647.7 N at 750 min^−1^. This relationship explains the strong influence of shaft speed on grinding intensity and simultaneously demonstrates why the chambers, shafts, bearings, crank elements, connecting plates, and main supports are exposed to rapidly increasing cyclic loads at higher operating speeds.

During the experimental regime, 50 kg of mineral feed was loaded into one chamber together with four cylindrical grinding bodies. The total grinding-body mass was

(29) $$m_{b,total}=4\times 7=28\;\mathrm{kg}$$

Therefore, the total internal moving mass in the loaded chamber was

$$m_{int}=m_{f}+m_{b,total}=50+28=78\;\mathrm{kg}$$

The ideal inertia-force amplitude associated with the 50 kg mineral feed is

(30) $$\begin{array}{c} F_{f}=m_{f}a_{c}\\ F_{f}=50\times 92.53=4626.5\;N \end{array}$$

The total inertia-force amplitude associated with the four grinding bodies is

$$F_{b,total}=28\times 92.53=2590.8\;N$$

The combined ideal inertia-force amplitude acting through the loaded chamber is therefore

(31) $$\begin{array}{c} F_{int}=m_{int}a_{c}\\ F_{int}=78\times 92.53=7217.3\;N \end{array}$$

The total gravitational force of the feed and grinding bodies is

$$F_{g,int}=78\times 9.81=765.2\;N$$

Depending on the angular position of the crank, gravity may act in the same direction as or opposite to the chamber-induced inertia force. The approximate extreme resultant loads are therefore

(32) $$F_{max}=F_{int}+F_{g,int}=7217.3+765.2=7982.5\;N$$

and

$$F_{min}=F_{int}-F_{g,int}=7217.3-765.2=6452.1\;N$$

Accordingly, the loaded chamber is subjected to an ideal rotating internal force with an amplitude of approximately 7.22 kN. The instantaneous resultant may vary between approximately 6.45 and 7.98 kN, depending on the relative direction of gravity and chamber acceleration. These values do not include the moving mass of the chamber, cover, support shafts, crank components, or connecting plates. They also exclude short-duration impact peaks generated by collisions between the grinding bodies and mineral fragments.

The 50 kg feed should not be treated as a completely rigid mass because the particles redistribute continuously inside the chamber. Nevertheless, the calculated value of 4.63 kN provides a conservative estimate of the maximum inertia load associated with the feed when it is accelerated together with the chamber. Since only one chamber was loaded, this force also represents the principal asymmetric load transferred through the chamber manufactured from 40X steel, the crankshaft, bearings, parallelogram linkage, main supports, and welded frame.

The grinding bodies also circulate relative to the internal cylindrical surface. The effective circulation radius is approximated as

(33) $$R_{b}=\frac{D_{c}-d_{b}}{2}$$

where $D_{c}$ is the internal working diameter of the chamber and $d_{b}$ is the grinding-body diameter. For

(34) $$D_{c}=140\;\mathrm{mm},d_{b}=45\;\mathrm{mm}$$

the circulation radius is

$$R_{b}=\frac{140-45}{2}=47.5\;\mathrm{mm}=0.0475\;m$$

The average relative angular velocity of the grinding-body system is determined from the experimentally observed circulation speed:

(35) $$\omega_{b}=\frac{2\pi n_{b}}{60}\;$$

For

$$\begin{array}{c} n_{b}=170\;\min^{-1}\\ \omega_{b}=\frac{2\pi \times 170}{60}=17.80\;\mathrm{rad}/s \end{array}$$

The radial force required to maintain the relative circular movement of one grinding body is

(36) $$F_{r}=m_{b}\omega_{b}^{2}R_{b}$$

Substitution of the selected parameters gives

$$F_{r}=7\times 17.80^{2}\times 0.0475\approx 105.4\;N$$

This value is approximately 1.53 times greater than the gravitational force acting on one grinding body. It characterises the radial force associated with relative circulation along the chamber wall and provides an estimate of the loading contributing to compression and abrasion of particles within the wall-contact zone.

However, $F_{r}\;$ should not be interpreted as an additional independent external centrifugal force. The actual normal reaction between the grinding body and chamber wall depends on the combined radial components of gravity, chamber acceleration, friction, mineral-bed resistance, impact forces, and the centripetal acceleration required for relative circulation.

Accordingly, the movement of one grinding body in the chamber-attached reference frame can be expressed as

(37) $$m_{b}a_{rel}=m_{b}g+N+F_{f}+F_{m}-m_{b}a_{c}$$

where $a_{rel}$ is the acceleration relative to the chamber, N is the normal wall reaction, $F_{f\;}$ is the friction force, $F_{m}$ represents resistance from the mineral material, and $-m_{b}a_{c\;}$ is the inertia force associated with chamber translation.

Resolving the equation into radial and tangential components provides the basis for describing the circulation of the grinding bodies. The tangential equation may be written in general form as

(38) $$m_{b}a_{t}=F_{c,t}+F_{g,t}-F_{f}-F_{m}$$

Because the direction of the chamber-induced inertia force rotates with the crankshaft, its radial and tangential components change continuously. At certain angular positions, the tangential component accelerates a grinding body along the chamber wall. At other positions, the force opposes its relative movement or increases the normal contact pressure. This behaviour explains why the average grinding-body circulation speed of 170 min^−1^ is substantially lower than the shaft speed of 750 min^−1^.

The kinetic energy associated with the relative motion of one grinding body is

(39) $$E_{k}=\frac{1}{2}m_{b}v_{b}^{2}$$

where the circumferential velocity is

(40) $$v_{b}=\omega_{b}R_{b}$$

For the investigated regime,

$$v_{b}=17.80\times 0.0475=0.846\;m/s$$

Therefore,

(41) $$E_{k}=\frac{1}{2}\times 7\times 0.846^{2}\approx 2.51\;J$$

per grinding body. For four grinding bodies, the total kinetic energy associated with their ideal relative circulation is approximately

$$E_{k,total}=4\times 2.51=10.04\;J$$

This energy is transferred repeatedly through grinding-body–particle, grinding-body–wall, and particle–particle contacts. The analytical results therefore confirm that the mill operates in a dynamically activated regime. The chamber-induced acceleration is approximately 9.43 times gravity, the loaded chamber develops an ideal internal inertia-force amplitude of approximately 7.22 kN, and the circulating grinding bodies provide repeated impact, compression, abrasion, and shear actions required for progressive fine and ultrafine comminution.

### 2.4. Finite Element Model

Finite element analysis was conducted to evaluate the strength, stiffness, and load-transfer behaviour of the double-chamber centrifugal-gyratory mill. The numerical investigation was performed in Autodesk Fusion using linear static stress analysis. Two complementary models were considered: a global model of the complete mill assembly and a local model of the shaft–chamber–support subsystem. The global model was used to assess the overall rigidity and structural load path of the machine, whereas the local model was used to identify stress concentrations and deformation in the mechanically critical drive region.

The investigated mill is driven by a single 3 kW electric motor operating at a nominal shaft speed of 750 min^−1^. The first cylindrical chamber is actuated by the primary crankshaft, while the second chamber is connected through two parallel plates and crank elements forming a parallelogram mechanism. During the experimental regime, 50 kg of mineral feed and four cylindrical grinding bodies with a total mass of 28 kg were placed in one chamber. The second chamber was mechanically connected but was not loaded with mineral material. The original assembly drawing specifies a nominal shaft speed of 750 min^−1^ and a feed capacity of approximately 50 kg per chamber. 

The numerical simulations were interpreted as static structural verification models rather than complete representations of the actual transient grinding process. The real machine is subjected to rotating inertia forces, cyclic bearing reactions, impacts from the grinding bodies, material redistribution, and possible dynamic amplification. These time-dependent effects cannot be reproduced completely using a linear static analysis. Consequently, the finite element results were used primarily to evaluate the structural reserve and identify potentially critical regions [37].

#### 2.4.1. Operational Load Definition

The nominal torque generated by the 3 kW motor at 750 min^−1^ was calculated as

(42) $$T_{m}=\frac{9550P}{n},$$

where P is the motor power in kW and n is the shaft speed in min^−1^. Therefore,

$$T_{m}=\frac{9550\times 3}{750}=38.2\;N\backslash \mathrm{cdotpm}.$$

This value represents the nominal continuous motor torque before considering starting torque, transmission efficiency, mechanical resistance, or short-duration overload.

The operating inertia load was calculated from the mass placed inside the loaded chamber. The chamber contained 50 kg of mineral feed and four grinding bodies, each with a mass of approximately 7 kg. The total internal mass was therefore

(43) $$m_{int}=50+4\times 7=78\;\mathrm{kg}.$$

For a crank eccentricity of 15 mm and a shaft speed of 750 min^−1^, the angular velocity and chamber acceleration were

(44) $$\omega_{c}=\frac{2\pi \times 750}{60}=78.54\;\mathrm{rad}/s,$$

$$a_{c}=e\omega_{c}^{2}=0.015\times 78.54^{2}=92.53\;{m/s}^{2}.$$

The ideal inertia-force amplitude generated by the feed and grinding bodies was consequently

(45) $$F_{int}=m_{int}a_{c}=78\times 92.53=7217\;N.$$

The gravitational load of the same internal mass was

(46) $$F_{g}=78\times 9.81=765\;N.$$

Depending on the angular position of the crank, gravity may act in the same direction as or opposite to the chamber-induced inertia force. The ideal resultant load therefore varies approximately between

(47) $$F_{min}=7217-765=6452\;N,$$

and

(48) $$F_{max}=7217+765=7982\;N.$$

Thus, the loaded chamber produces an ideal rotating internal force with an amplitude of approximately 7.22 kN. This value does not include the inertia of the chamber shell, cover, crank supports, shafts, connecting plates, or other moving structural components. It also excludes short-duration impact loads generated when the grinding bodies collide with mineral fragments or the chamber wall.

Because only one chamber contained 50 kg of feed, the experimental condition produced an asymmetric dynamic load. The inertia force generated solely by the mineral feed was

(49) $$F_{f}=50\times 92.53=4627\;N.$$

This asymmetric force is transferred through the 40X steel chamber, crankshafts, bearing supports, parallelogram connecting plates, main supports, and welded frame. In a physically representative transient model, the calculated force should be applied as a rotating harmonic load or evaluated at several crank positions, such as 0°, 90°, 180°, and 270°.

The existing Fusion simulations used much larger torsional moments than the nominal motor torque. Therefore, the 500 and 900 N·m moments were interpreted as amplified static overload cases rather than as direct representations of the 3 kW motor.

#### 2.4.2. Global Mill Model

The global model was developed from the complete three-dimensional assembly of the double-chamber centrifugal-gyratory mill. It included the welded base frame, two parallel cylindrical chambers, main supports, side plates, crankshaft assemblies, connecting elements, bearing-support regions, shaft components, and principal drive-side parts. The two chambers were represented as mechanically connected through the parallelogram linkage.

Small geometric features that did not substantially influence the global load path were removed or simplified to reduce computational cost. The main purpose of the model was to reproduce the transfer of loading from the crank and chamber-support assemblies through the side supports and into the common frame.

The analysis was performed in Autodesk Fusion version 2703.1.11 using a linear static stress study. The lower supporting surfaces of the frame were constrained using fixed boundary conditions. Translation was prevented in the global x, y, and z directions, representing rigid attachment of the machine to a stiff foundation or welded supporting platform.

The fixed-base assumption provides a repeatable reference condition but idea lises the actual installation. It does not represent flexibility of the foundation, anchor-bolt deformation, local movement between the frame and floor, or compliance of welded and bolted connections. The calculated global displacement may therefore be lower than the displacement occurring in the physical prototype [38,39].

Three torsional moments of 900 N·m were applied about the longitudinal shaft axis at selected torque-transfer regions. The original model used these moments to reproduce severe simultaneous loading of the crank and chamber-support system. 

For the revised interpretation, these moments should not be described as the normal torque generated by the 3 kW motor. Each 900 N·m moment is approximately

(50) $$\frac{900}{38.2}\approx 23.6$$

times greater than the calculated nominal motor torque. The global study therefore represents an artificially amplified structural load intended to examine the strength reserve under conditions such as jamming, impact-related torque peaks, start-up shock, or extreme mechanical resistance.

The three applied moments should also not automatically be summed and interpreted as a single 2700 N·m motor torque. They act at different model regions and constitute a numerical loading scheme used to examine the structural load path of the assembly.

#### 2.4.3. Local Shaft–Chamber–Support Model

A second finite element model was developed to investigate the local response of the principal drive subsystem. The reduced model included one cylindrical grinding chamber, an extended shaft, a hub or wheel element, side brackets, support members, and the adjacent base structure.

The local model was analysed in Autodesk Fusion version 2704.1.15 using the same linear static solution procedure. The lower support surfaces were fixed in all translational directions. A single torsional moment of 500 N·m was applied to the selected shaft surface about its longitudinal axis. 

The applied torque was approximately

(51) $$\frac{500}{38.2}\approx 13.1$$

times greater than the nominal torque of the 3 kW motor. Therefore, the local simulation also represents a severe static overload condition rather than normal continuous operation.

The reduced model provided a more direct load path through the shaft and chamber supports and did not include the full stiffness contribution of the complete two-chamber frame. It was therefore suitable for identifying local deformation and stress concentrations caused by combined torsion, bending, shear, and support reactions. Particular attention was given to:

- Shaft-diameter transitions;
- Hub and shaft interfaces;
- Bearing-support regions;
- Side brackets;
- Chamber-support connections;
- Changes in structural cross-section.

The local and global models were not treated as alternative mill designs because their geometries, constraints, and applied moments were different. Instead, they were interpreted as complementary models representing different structural scales.

#### 2.4.4. Material Properties

The grinding chambers and principal shafts of the laboratory mill were manufactured from 40X chromium-alloy structural steel. 40X steel is comparable in chemical composition and mechanical properties to European chromium-alloy structural steels and is considered suitable for this application. The selection of this material is based on the fact that these components are subjected to cyclic torsion, bending, impact loading, contact pressure, and abrasive interaction with the processed mineral material [40].

Repeated grinding-body impacts;

- Distributed pressure from the mineral bed;
- Abrasive wear of the internal surface;
- Cyclic reactions from the crank supports.
- The 40X steel shaft must withstand:
- Motor torque;
- Alternating bending;
- Eccentric inertia loading;
- Bearing reactions;
- Stress concentrations at shoulders and fitted surfaces.

However, the existing Autodesk Fusion reports assigned the same generic steel material to the load-bearing components. The material model was linear elastic and isotropic, with the following properties: The material model was linear elastic and isotropic, with the assigned material properties summarized in Table 2.

These values were retained in the reported simulations to provide a consistent common yield criterion.

The existing results should therefore be interpreted relative to the assigned generic steel properties, not as a complete material-specific verification of heat-treated 40X steel. The mechanical properties of 40X depend strongly on heat treatment, hardness, section thickness, and manufacturing condition. A final design study should assign certified 40X properties separately to the chambers and shafts while retaining appropriate structural-steel properties for the welded frame and support plates [41].

#### 2.4.5. Contact Conditions and Mesh

The connected components were represented using bonded contacts. This formulation prevents sliding and separation between mating surfaces and provides a stable solution for a large assembly containing numerous interfaces. Bonded contact is suitable for preliminary representation of welded joints and rigidly connected components.

However, this assumption idealises the behaviour of:

- Bearings;
- Shaft–hub fits;
- Bolted connections;
- Removable chamber covers;
- Fitted crank components;
- Interfaces with clearance.

Bonded contacts generally increase the calculated stiffness compared with a real machine. Therefore, the models are suitable for preliminary global strength evaluation but not for detailed prediction of bearing behaviour, bolt loading, slip, or local separation.

Both models were discretised using second-order parabolic solid elements. The global mesh contained 130,900 nodes and 66,670 elements. The local mesh contained 130,367 nodes and 66,416 elements. The average element size was defined as 10% of the corresponding model size, the maximum adjacent element-size ratio was 1.5, and the maximum permitted aspect ratio was 10. No adaptive refinement steps were performed.

Parabolic elements provide an improved representation of curved surfaces, shaft transitions, and bending compared with first-order tetrahedral elements. Nevertheless, the absence of mesh convergence testing means that local peak stresses at sharp transitions should be interpreted cautiously. Additional refinement is required at the shaft shoulders, bearing seats, support-bracket fillets, crank connections, and welded frame transitions [42,43].

#### 2.4.6. Evaluation Criteria

Both models were evaluated using equivalent von Mises stress, principal stresses, directional normal and shear stresses, total displacement, directional displacement, equivalent strain, reaction forces, contact forces, contact pressure, and minimum safety factor.

The von Mises criterion was used as the principal strength indicator because the chamber, shaft, supports, and frame were modelled as ductile metallic components subjected to multiaxial loading. The static safety factor was calculated as

(52) $$SF=\frac{\sigma_{y}}{\sigma_{vM}},$$

where $\sigma_{y}$ is the assigned material yield strength and $\sigma_{vM}$ is the local equivalent von Mises stress.

Total displacement was used to assess whether the supporting structure could maintain shaft alignment, parallel chamber orientation, and crank-linkage geometry. Reaction forces were examined to determine how the applied loads were transferred to the fixed frame supports, while contact pressure was used to identify interfaces requiring more detailed local analysis [44].

The present finite element models provide a preliminary assessment of static structural capacity. They do not directly reproduce the rotating 7.22 kN internal inertia load, grinding-body impacts, cyclic fatigue, bearing clearances, or vibration of the one-loaded-chamber configuration. A subsequent model should therefore combine the analytically derived chamber load with transient dynamic, modal, and fatigue analyses.

## 3. Results

### 3.1. Grinding Performance

The grinding performance of the double-chamber centrifugal-gyratory mill was evaluated under operating conditions selected to generate intensive interaction between the grinding bodies, mineral feed, and internal chamber surface. The physical tests demonstrated that the proposed mechanism was capable of fracturing mechanically resistant mineral fragments and producing a visibly increased proportion of smaller particles. During operation, the two horizontal cylindrical chambers performed synchronous plane-parallel circular motion generated by a common crank mechanism. The chambers were mechanically connected by two parallel linkage plates forming a parallelogram-type system, which maintained their parallel orientation and ensured coordinated motion from a single drive.

The mill was driven by one 3 kW electric motor operating at a nominal shaft speed of approximately 750 min^−1^. Mechanical power was transmitted to the primary crankshaft, while the movement of the first chamber was transferred to the second chamber through the parallelogram crank linkage. Unlike a conventional drum mill, the cylindrical chambers did not rotate continuously around their longitudinal axes. Instead, the complete chamber bodies moved along circular trajectories determined by the crank eccentricity.

During the investigated experiment, 50 kg of hard mineral feed was loaded into one chamber. Four cylindrical grinding bodies, each with a mass of approximately 7 kg and a diameter of 45 mm, were installed in the same chamber. The total grinding-body mass was therefore

(53) $$m_{b}=4\times 7=28\;\mathrm{kg},$$

and the total internal moving mass was

(54) $$m_{int}=50+28=78\;\mathrm{kg}.$$

The second chamber remained mechanically connected and moved synchronously, but it was not loaded with mineral feed during the investigated test. This arrangement created an asymmetric operating condition and allowed the structural response of the mill to be evaluated under a more severe unbalanced load.

For the nominal crank speed of 750 min^−1^ and a crank eccentricity of 15 mm, the crank angular velocity was

(55) $$\omega_{c}=\frac{2\pi n_{c}}{60}=\frac{2\pi \times 750}{60}=78.54\;\mathrm{rad}/s.$$

The corresponding chamber-centre acceleration was

(56) $$a_{c}=e\omega_{c}^{2}=0.015\times 78.54^{2}=92.53\;{m/s}^{2}.$$

This acceleration is approximately 9.43 times greater than gravitational acceleration. The inertia-force amplitude acting on one 7 kg grinding body was

(57) $$F_{b}=m_{b}a_{c}=7\times 92.53=647.7\;N.$$

The corresponding gravitational force was only 68.67 N. Therefore, the movement and wall contact of the grinding bodies were controlled primarily by chamber-induced inertia rather than by gravity.

The ideal inertia-force amplitude associated with the 50 kg feed was

(58) $$F_{f}=50\times 92.53=4626.5\;N.$$

The total inertia-force amplitude generated by the four grinding bodies was

(59) $$F_{b,total}=28\times 92.53=2590.8\;N.$$

The combined ideal internal inertia load in the filled chamber was consequently

(60) $$F_{int}=78\times 92.53=7217.3\;N.$$

Thus, the chamber was subjected to a rotating internal load with an ideal amplitude of approximately 7.22 kN. Depending on the angular relationship between gravity and chamber acceleration, the instantaneous resultant load varied theoretically between approximately 6.45 and 7.98 kN. These values exclude the moving mass of the chamber, cover, crank supports, shafts, and connecting plates, as well as short-duration impact peaks.

The grinding bodies circulated relative to the internal chamber surface at an experimentally estimated average speed of approximately 170 min^−1^. For an effective circulation radius of 47.5 mm, the relative circumferential velocity was

(61) $$v_{b}=0.846\;m/s,$$

and the corresponding kinetic energy was

(62) $$E_{k}=\frac{1}{2}m_{b}v_{b}^{2}\approx 2.51\;J$$

per grinding body. Four grinding bodies therefore possessed a combined ideal relative kinetic energy of approximately 10.04 J. This energy was transferred repeatedly through grinding-body–particle, grinding-body–wall, and particle–particle contacts.

The experimental observations showed that coarse mineral fragments were initially fractured by direct impact and concentrated compression. Particles entering the narrowing contact region between a grinding body and the chamber wall were subjected to high local pressure. Sliding and rolling of the grinding bodies generated additional abrasion and shear, while continuous movement of the feed produced interparticle collisions and self-grinding.

The single-motor crank-linked configuration maintained coordinated movement of both chambers without the speed and phase differences associated with independently driven chambers. The 3 kW motor operated without visible interruption during the test, and the crank mechanism maintained the required plane-parallel chamber motion. No visible permanent deformation of the principal supports, chambers, shafts, or connecting plates was detected after operation.

The chambers and principal shafts were manufactured from 40X chromium-alloy structural steel. This material was selected because these components experience repeated impact, bending, torsion, contact pressure, and abrasive loading. The strength, hardenability, and wear resistance of 40X steel make it more suitable for the mechanically critical chamber and shaft components than ordinary low-carbon structural steel.

The obtained results demonstrate that the available motor power was successfully converted into cyclic chamber motion and intensive grinding-body interaction. However, the present tests confirm hard-rock fracture and progressive particle-size reduction only. Quantitative classification of the product as fine or ultrafine requires measured particle-size distribution and specific energy-consumption data.

### 3.2. Particle-Size Reduction

The experimental grinding trial produced a clear transformation of the initial hard-rock feed into a heterogeneous product containing fractured coarse particles and a visibly increased proportion of smaller fragments. The observed reduction in particle size confirms that the plane-parallel circular motion of the chamber generated sufficiently intensive mechanical interaction to overcome the fracture resistance of the processed mineral material.

At the beginning of the grinding cycle, the feed consisted mainly of relatively large and mechanically resistant mineral fragments. These particles were exposed to repeated loading from four cylindrical grinding bodies operating inside the filled chamber. Because the chamber-generated acceleration was approximately 9.43 times greater than gravity, the grinding bodies remained intensively pressed against the mineral bed and internal chamber surface throughout a substantial part of the operating cycle.

The particle-size reduction process occurred through several complementary mechanisms. Direct impact was particularly important during the initial destruction of the largest fragments. When a grinding body collided with a mineral particle, the short-duration contact generated local tensile, compressive, and shear stresses. Existing microcracks, pores, grain boundaries, and mineralogical discontinuities acted as preferential fracture paths. Repeated loading caused these defects to propagate until the original fragments separated into smaller particles.

Compression occurred when particles entered the contact region between a cylindrical grinding body and the chamber wall. The chamber-induced force acting on one grinding body reached approximately 647.7 N at 750 min^−1^. Particles trapped within the narrowing contact region were therefore subjected to concentrated local loading that considerably exceeded the loading produced by the weight of the grinding body alone.

After the initial coarse fragments were broken, abrasion and shear became increasingly important. Smaller particles were transported within the material layer between the grinding bodies and chamber surface. Relative sliding and rolling produced surface damage, edge rounding, and progressive reduction in particle dimensions. At the same time, particles moving at different velocities repeatedly collided with one another and were compressed within the circulating material bed.

Interparticle grinding reduced the dependence of the process on direct steel-to-steel contact and contributed to the formation of the fine fraction. The continuous change in the direction of chamber acceleration redistributed the mineral feed and repeatedly returned particles to active impact and compression zones. As a result, fragments that were not broken during one loading event were exposed to further loading during subsequent crank revolutions.

Although the machine contains two mechanically connected chambers, only one chamber was loaded with 50 kg of mineral material during the investigated experiment. Therefore, the reported particle-size reduction relates to the operation of one active grinding chamber rather than simultaneous processing in both chambers. The second chamber contributed to the kinematics and structural reactions of the mechanism but did not increase the processed feed mass during this test.

Visual comparison of the initial feed and ground product confirmed fracture of the original mineral fragments and formation of smaller particles. However, visual observations alone are insufficient to determine the final product classification. Quantitative evaluation should include sieve analysis or laser-diffraction measurement of parameters such as $d_{10}$, $d_{50}$, $d_{80}$, and $d_{90}$.

The reduction ratio can be expressed as

(63) $$R_{80}=\frac{d_{80,feed}}{d_{80,product}},$$

where $d_{80,feed}\;$ and $d_{80,product}\;$ are the particle sizes at which 80% of the feed and product pass, respectively.

The present experimental observations demonstrate the practical breakage capability of the mill and support the analytical conclusion that centrifugal-gyratory loading intensifies particle destruction. Nevertheless, measured particle-size distributions, repeated trials, mass recovery, grinding time, and energy consumption are required for a complete quantitative evaluation.

### 3.3. Structural Analysis

The structural performance of the double-chamber centrifugal-gyratory mill was evaluated using two complementary static stress analyses performed in Autodesk Fusion. The first model represented the complete mill assembly and was used to examine the global structural behaviour of the two chambers, frame, chamber supports, crank components, connecting plates, and principal shaft elements. The second model represented a reduced shaft–chamber–support subsystem and was used to investigate local stresses, displacement, and contact loading in the drive-side region.

The two analyses were conducted separately because the complete machine and the reduced shaft subsystem represent different structural scales. The global model characterises the overall stiffness and load-transfer behaviour of the mill, whereas the local model provides a more detailed representation of the shaft, hub, chamber support, and adjacent brackets.

The actual machine is driven by a single 3 kW motor. At 750 min^−1^, the nominal continuous motor torque is

(64) $$T_{m}=\frac{9550P}{n}=\frac{9550\times 3}{750}=38.2\;N\backslash \mathrm{cdotpm}.$$

The 500 and 900 N·m moments used in the existing finite element studies are therefore substantially greater than the nominal operating torque. They were interpreted as amplified static overload cases representing possible jamming, start-up resistance, impact-related torque peaks, or extreme material resistance rather than continuous motor loading.

The experimental configuration also generated an asymmetric chamber load because 50 kg of mineral feed and 28 kg of grinding bodies were placed in only one chamber. The ideal internal inertia-force amplitude of this 78 kg mass was approximately 7.22 kN. This force rotated with the crank position and was transmitted through the 40X steel chamber, shaft, bearings, linkage plates, supports, and frame.

The finite element models used linear static stress analysis and therefore did not reproduce the time-dependent rotation of the 7.22 kN load, individual grinding-body impacts, cyclic bearing reactions, vibration, or fatigue. The results were used as preliminary indicators of structural strength and stiffness rather than as a complete dynamic representation of the grinding process.

The load-bearing parts in the existing Fusion models were assigned generic isotropic structural steel with a density of 7850 kg/m^3^, Young’s modulus of 210 GPa, Poisson’s ratio of 0.30, yield strength of 207 MPa, and ultimate tensile strength of 345 MPa. The physical chamber and shaft were selected as 40X steel, but the existing numerical results must be interpreted relative to the generic steel properties used in the simulations.

Second-order parabolic solid elements were used in both models. Bonded contacts were assigned between connected components, and the lower frame or support surfaces were fixed in all translational directions. The numerical results were evaluated using von Mises stress, principal stress, equivalent strain, total displacement, reaction force, contact force, contact pressure, and minimum safety factor.

#### 3.3.1. Main Cylindrical Mill Assembly

The global finite element model represented the complete double-chamber mill assembly. It included the two parallel cylindrical chambers, welded base frame, main supports, chamber brackets, crankshaft regions, linkage plates, connecting components, and structural elements responsible for transferring the operating loads to the foundation.

The model contained 130,900 nodes and 66,670 second-order parabolic solid elements. Three torsional moments of 900 N·m were applied at selected torque-transfer surfaces of the crank and chamber-support system. These moments represented severe amplified static loading rather than the nominal torque of the 3 kW motor.

Each 900 N·m moment was approximately

$$\frac{900}{38.2}=23.6$$

times greater than the nominal motor torque. Therefore, the load case was used to evaluate the structural reserve of the assembly under extreme torsional resistance. It should not be described as the normal continuous operating condition of the mill.

The global model did not explicitly include the rotating 7.22 kN inertia force generated by the 50 kg feed and four grinding bodies. For improved physical correspondence, a subsequent simulation should apply the analytically calculated force to the loaded chamber at several crank positions or use a transient dynamic analysis. Figure 6 shows the torsional moments applied to the chamber and crank-support regions of the global finite element model.

The maximum equivalent von Mises stress in the complete mill assembly was 22.915 MPa, corresponding to approximately 11.1% of the assigned steel yield strength of 207 MPa. The minimum safety factor was 9.033, indicating a considerable static strength reserve under the adopted amplified loading conditions. The highest stresses occurred near the chamber supports, crankshaft connections, side brackets, and transitions between the supports and base frame. 

The maximum first principal stress was 21.328 MPa, while the minimum third principal stress was −19.398 MPa, confirming combined tensile, compressive, bending, and torsional loading. The maximum total displacement was only 0.050 mm, demonstrating sufficient frame stiffness to maintain chamber and shaft alignment. The maximum reaction force was 2652.92 N, the maximum contact force was 2019.565 N, and the maximum contact pressure was 8.884 MPa. These results show that the frame provides a stable load path from the crank mechanism to the foundation.

#### 3.3.2. Crusher-Shaft Assembly

The second finite element model included one cylindrical chamber, the crusher shaft, hub, bearing-support brackets, and adjacent frame elements. The shaft is a critical component because it transfers torque from the single 3 kW motor to the eccentric crank mechanism while supporting the cyclic movement of the chamber. The reduced model contained 130,367 nodes and 66,416 parabolic solid elements. A torsional moment of 500 N·m was applied to the shaft as a conservative static overload case.

The maximum von Mises stress was 65.837 MPa, corresponding to approximately 31.8% of the assigned yield strength. The minimum safety factor was 3.144. Although the stress was almost three times higher than in the complete assembly, it remained below the yield limit. The results confirm that the shaft and support system can withstand the applied 500 N·m static torque without plastic deformation. Figure 7 Structural response of the shaft–chamber–support assembly under torque load.

The maximum total displacement in the crusher-shaft model was 0.740 mm. The greatest deformation occurred in the cylindrical chamber and the more distant sections of the shaft-support structure. This value was higher than the 0.050 mm displacement obtained for the complete mill because the reduced model had a longer and less supported load path. The applied torque produced both shaft torsion and local bending of the chamber supports and brackets.

The maximum reaction force was 6475.121 N, while the maximum contact force and contact pressure reached 5589.325 N and 44.975 MPa, respectively. These results identify the shaft shoulders, hub connection, bearing-support regions, and bracket transitions as the most highly loaded areas. Particular attention should therefore be given to shaft diameter, fillet radii, bearing-seat geometry, bracket thickness, weld quality, and support rigidity.

The two finite element studies collectively indicate adequate preliminary static strength. The complete assembly produced a maximum von Mises stress of 22.915 MPa and a displacement of 0.050 mm, whereas the local crusher-shaft model produced a maximum stress of 65.837 MPa and a displacement of 0.740 mm. In both cases, the calculated stresses remained below the assigned yield strength of 207 MPa. 

## 4. Discussion

The results demonstrate that the double-chamber centrifugal-gyratory mill operates according to a fundamentally different principle from conventional gravity-dominated drum mills. The two cylindrical chambers perform synchronous plane-parallel circular motion generated by a single 3 kW motor and a mechanically connected crank system. The parallelogram linkage maintains the parallel orientation of the chambers and transfers motion from the driven chamber to the second chamber.

At a shaft speed of 750 min^−1^ and a crank eccentricity of 15 mm, the chamber-centre acceleration reached 92.53 m/s^2^, corresponding to approximately 9.43 times gravitational acceleration. The inertia-force amplitude acting on one 7 kg grinding body was approximately 647.7 N. Therefore, the grinding process was governed mainly by chamber-induced inertial loading rather than by the weight of the grinding bodies.

During the experiment, 50 kg of hard mineral feed and four grinding bodies with a combined mass of 28 kg were loaded into one chamber. The total internal moving mass was therefore 78 kg, producing an ideal inertia-force amplitude of approximately 7.22 kN. This asymmetric loading condition was transferred through the 40X steel chamber, crankshaft, bearings, connecting plates, supports, and common frame.

The experimental observations supported the analytical model. Hard mineral fragments were reduced through the combined action of impact, compression, abrasion, shear, and interparticle interaction. Large particles were mainly fractured by direct impact and compression between the grinding bodies and chamber wall. As particle size decreased, abrasion and particle-to-particle grinding became increasingly important. The continuously changing direction of the inertia force promoted material circulation and repeatedly returned particles to active grinding zones.

The finite element results showed that the complete mill assembly had high global stiffness. The maximum equivalent stress was 22.915 MPa, the maximum displacement was 0.050 mm, and the minimum safety factor was 9.033. The local crusher-shaft model showed a higher stress of 65.837 MPa, a displacement of 0.740 mm, and a minimum safety factor of 3.144. The higher local response resulted from concentrated loading through the shaft, hub, bearing supports, and brackets. 

Although both models remained below the assigned yield strength, the applied moments of 500 and 900 N·m were substantially higher than the nominal 38.2 N·m torque of the 3 kW motor. These simulations should therefore be interpreted as amplified static overload cases rather than as representations of normal operating conditions.

Several limitations of the present study should be considered when interpreting these results. First, the structural analysis was performed under simplified static loading conditions and did not explicitly account for dynamic amplification associated with transient operation, resonance, or variations in rotational speed. Second, fatigue effects were not evaluated, although the shaft, crank mechanism, bearings, chamber supports, and connecting elements are subjected to repeated cyclic loading during mill operation. Third, the experimental loading was applied to only one chamber. Therefore, the experiment did not fully reproduce the coupled dynamic response of the two simultaneously operating chambers, including possible interactions between their inertial forces and vibrations. Fourth, the finite element models were based on linear elastic material behavior, simplified contact conditions, bonded connections, and idealized boundary constraints. Consequently, nonlinear contact behavior, friction, joint clearances, bearing compliance, and material plasticity were not explicitly represented. Finally, the transient impact forces generated by interactions among the grinding bodies, mineral particles, and chamber walls were not directly quantified. Their magnitude and time-dependent characteristics depend on grinding-body motion, filling conditions, material properties, particle distribution, and operating speed.

Overall, the analytical calculations, experimental observations, and structural simulations indicate that the proposed mill combines intensive mineral breakage with adequate preliminary structural strength. However, the present results should be regarded as an initial theoretical, experimental, and design-oriented assessment rather than a complete validation of the mill under full dynamic operating conditions. The shaft-support and bearing regions were identified as the most highly loaded structural areas in the present static FEA, while the complete frame demonstrated sufficient rigidity to maintain chamber alignment under the analyzed loading cases. Further work should include simultaneous two-chamber testing, transient dynamic analysis, fatigue-life assessment, experimental measurement of impact loads, quantitative particle-size distribution analysis, and specific energy-consumption evaluation.

## 5. Conclusions

This study presented the design, analytical assessment, experimental evaluation, and finite element verification of a double-chamber centrifugal-gyratory mill for the fine and ultrafine grinding of mineral raw materials. The developed mill uses a single 3 kW drive and a mechanically coupled double-chamber configuration to generate synchronous plane-parallel circular motion.

The analytical results confirmed the formation of an intensive inertia-based grinding regime. At a crank eccentricity of 15 mm and a shaft speed of 750 min^−1^, the chamber acceleration reached 92.53 m/s^2^, corresponding to approximately 9.43 g. The resulting inertia force acting on a 7 kg grinding body was approximately 647.7 N. For the investigated 78 kg internal moving mass, the calculated ideal inertia-force amplitude reached approximately 7.22 kN. Experimental observations confirmed the fracture of mechanically resistant mineral material and progressive particle-size reduction through combined impact, compression, abrasion, shear, and interparticle interactions.

Finite element analysis demonstrated adequate static structural capacity under the investigated amplified loading conditions. In the global model, the maximum von Mises stress and displacement were 22.915 MPa and 0.050 mm, respectively, with a minimum safety factor of 9.033. In the local shaft–chamber–support model, the corresponding values were 65.837 MPa, 0.740 mm, and 3.144. The calculated stresses remained below the assigned steel yield strength of 207 MPa. Since the applied torques of 500 and 900 N·m substantially exceeded the nominal motor torque of approximately 38.2 N·m, these results should be interpreted as conservative static overload assessments rather than representations of normal operating conditions.

Overall, the results demonstrate the technical feasibility of the proposed double-chamber centrifugal-gyratory mill and confirm its ability to generate intensive grinding action while maintaining adequate static structural strength. However, the present experiments demonstrate hard-rock fracture and progressive particle-size reduction rather than quantitatively confirming fine or ultrafine product specifications. Such confirmation requires direct measurement of particle-size distribution, throughput, and specific energy consumption.

Future work will therefore focus on quantitative particle-size analysis, experimental determination of specific energy consumption and throughput, and optimization of the main operating and design parameters. Further investigations will also include transient dynamic, modal, and fatigue analyses, experimental vibration measurements, grinding-media wear assessment, and evaluation of processing costs. These studies will provide the quantitative basis required to assess the energy efficiency, durability, and industrial applicability of the proposed mill.

## Figures and Tables

**Figure 1:**
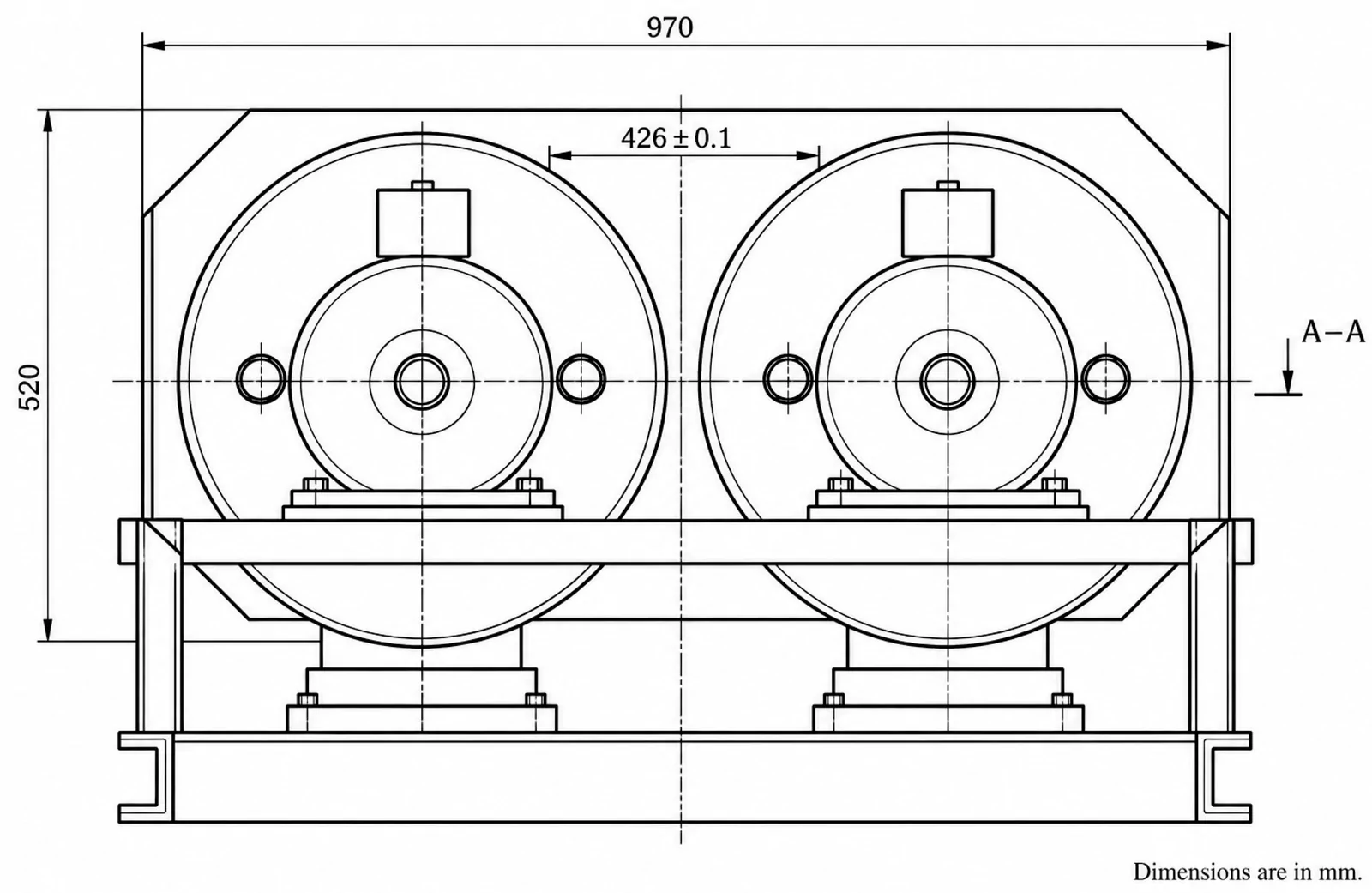
Front view of the double-chamber centrifugal-gyratory mill with principal dimensions.

**Figure 2:**
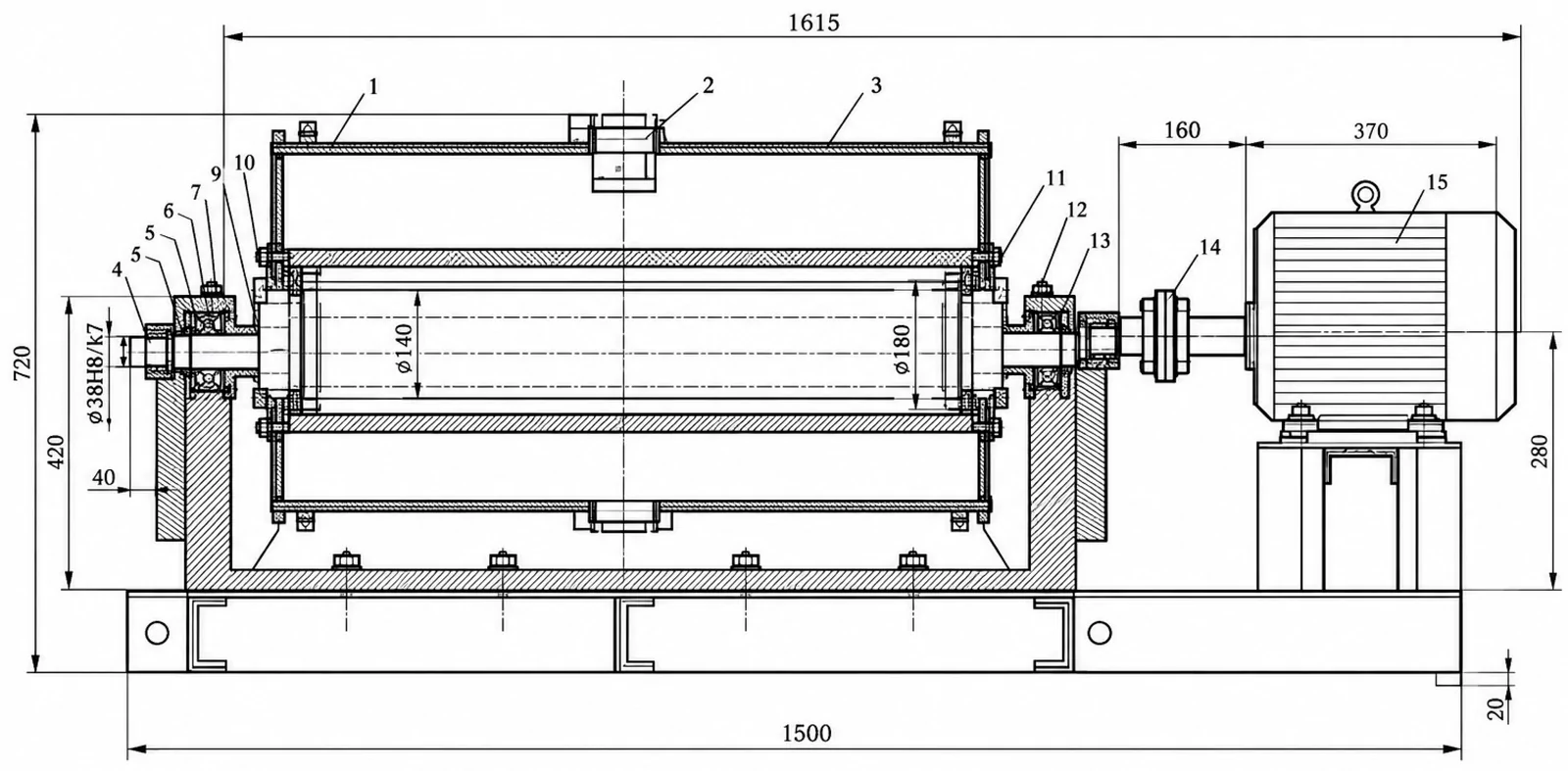
Side view of the double-chamber centrifugal-gyratory mill showing the grinding chambers, supporting structure, shaft system, and drive unit.

**Figure 3:**
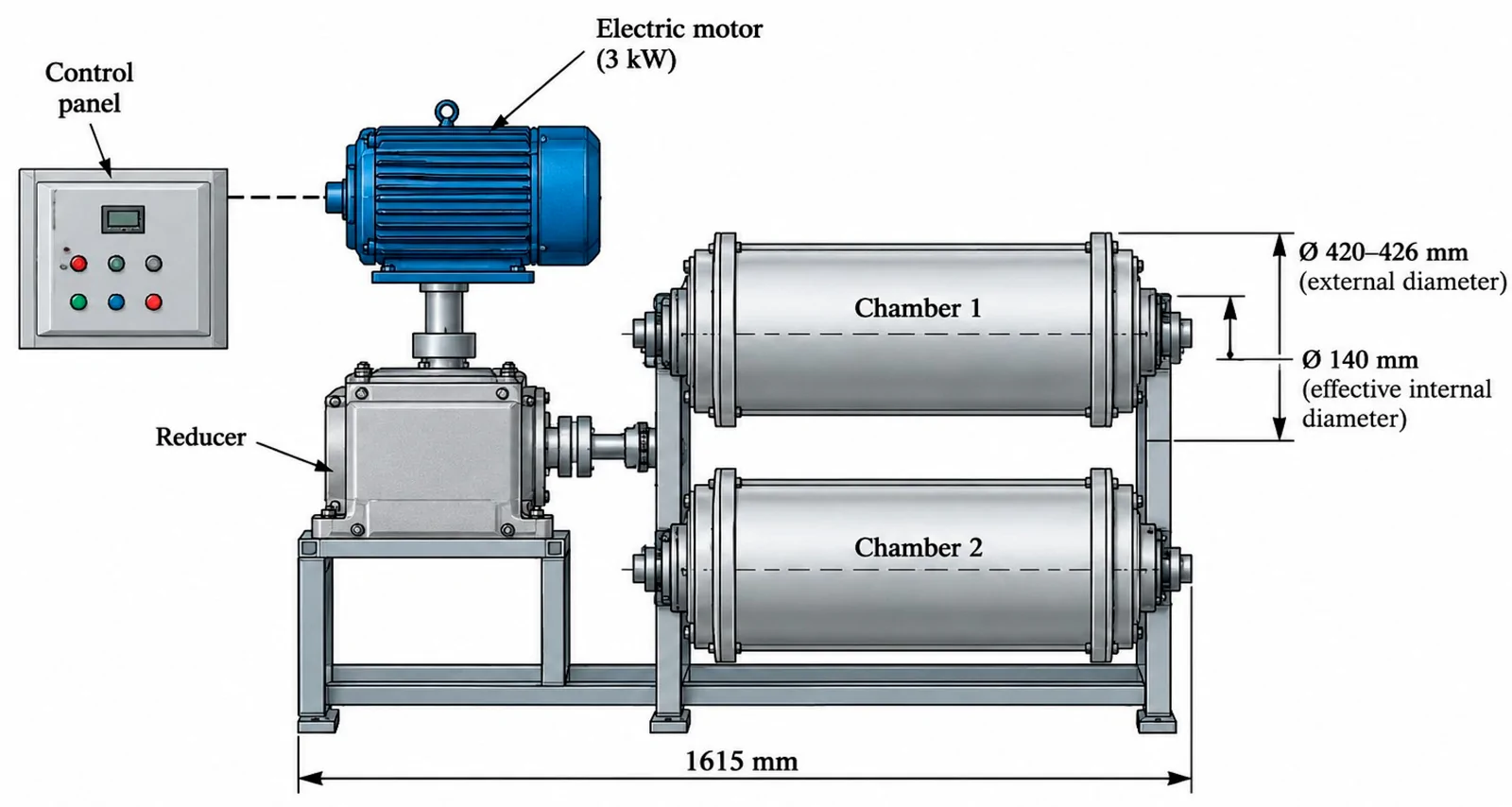
Functional scheme of the double-chamber centrifugal-gyratory mill showing the control panel, electric motor, reduction unit, drive system, and grinding chambers.

**Figure 4:**
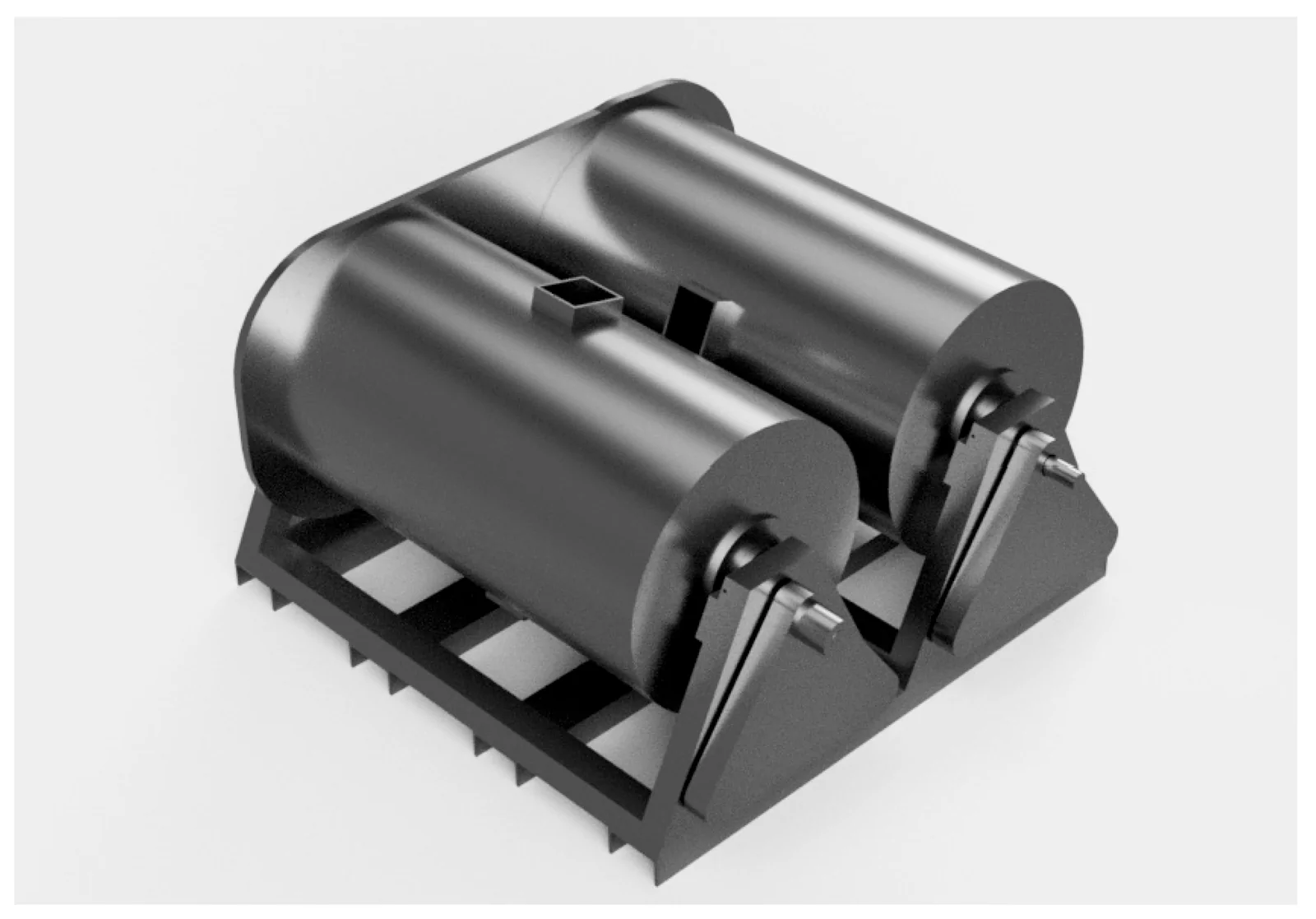
3D design of two chamber mill.

**Figure 5:**
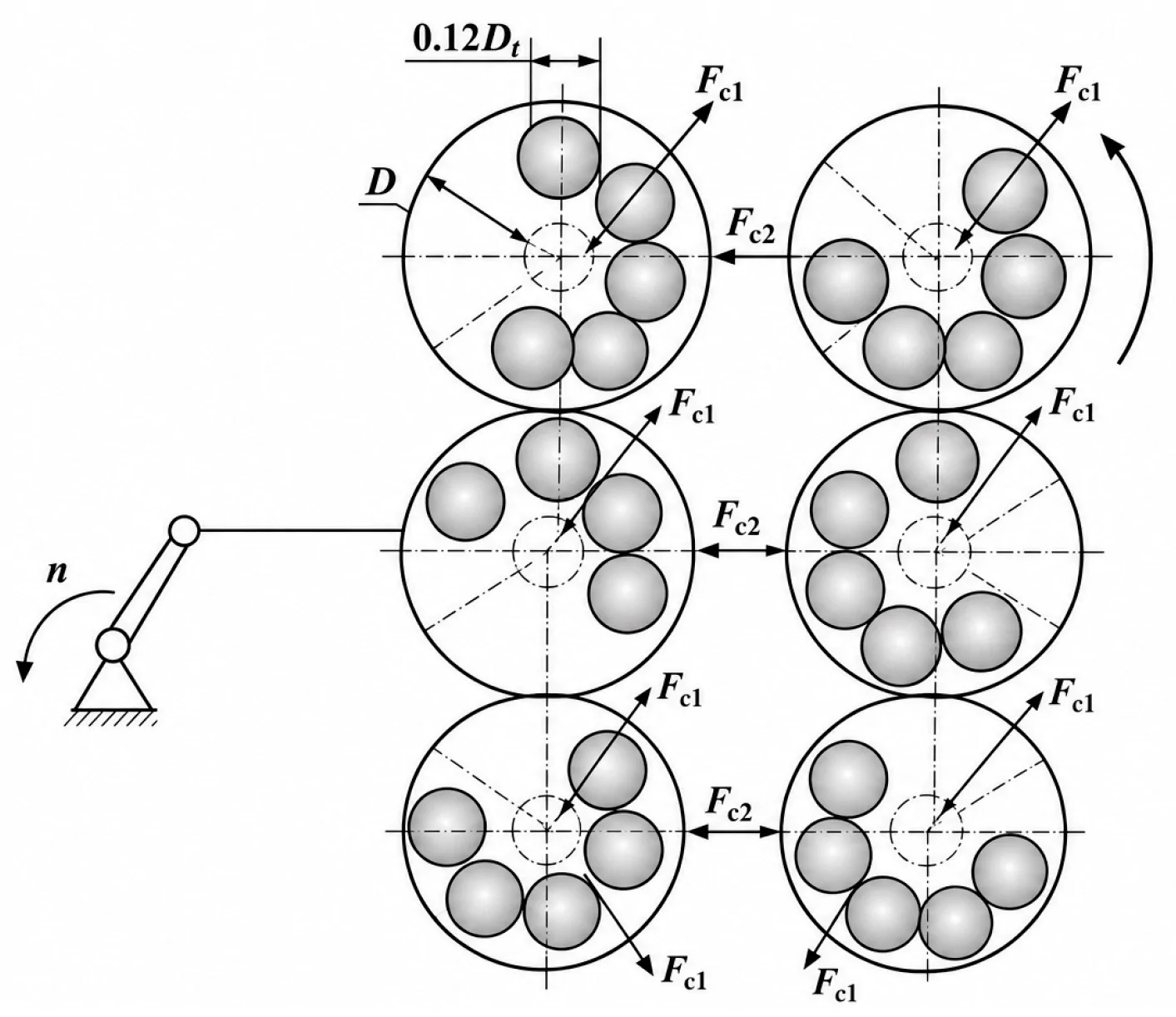
Kinematic scheme of two chamber mill.

**Figure 6:**
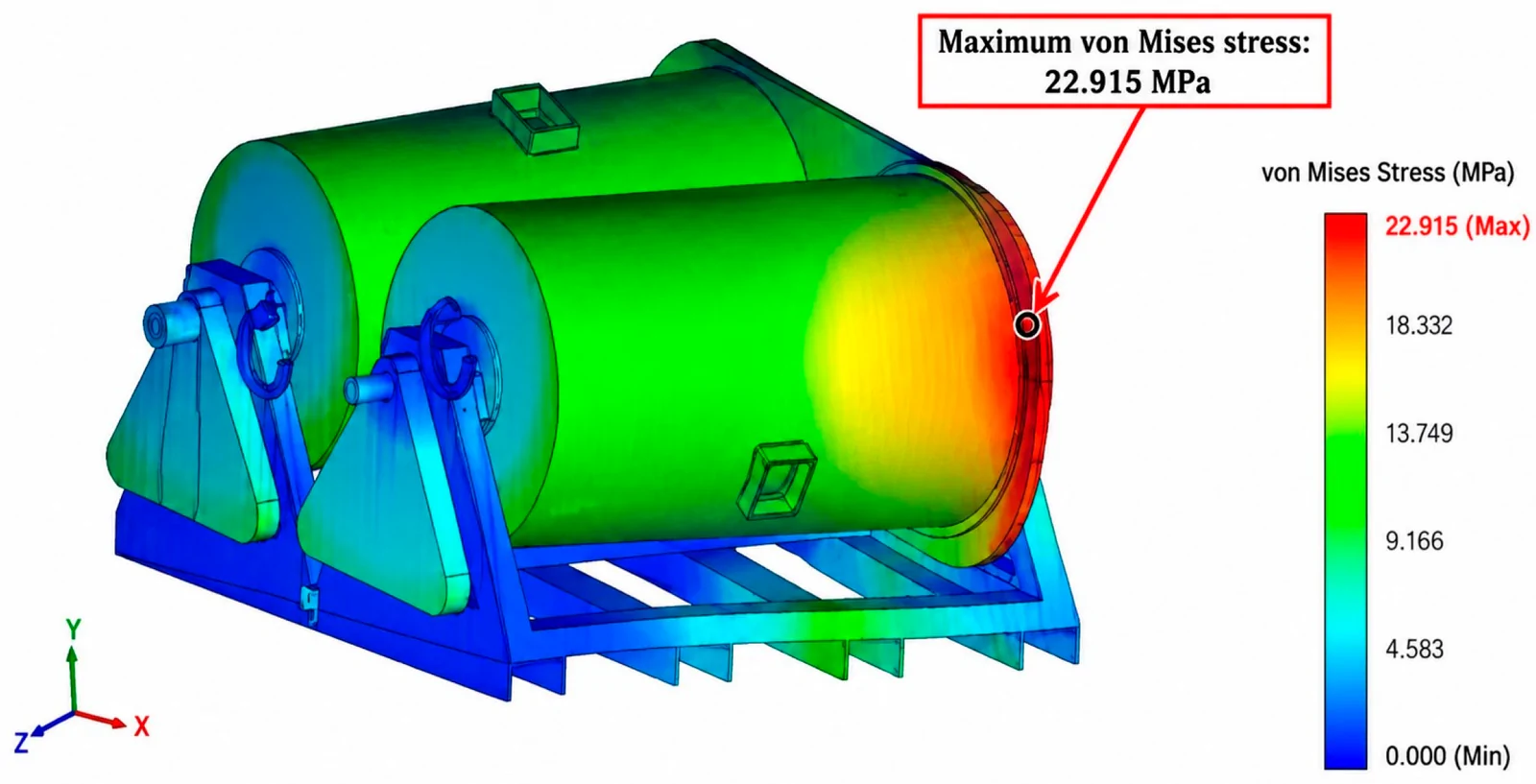
Structural response of the complete mill assembly under torque load.

**Figure 7:**
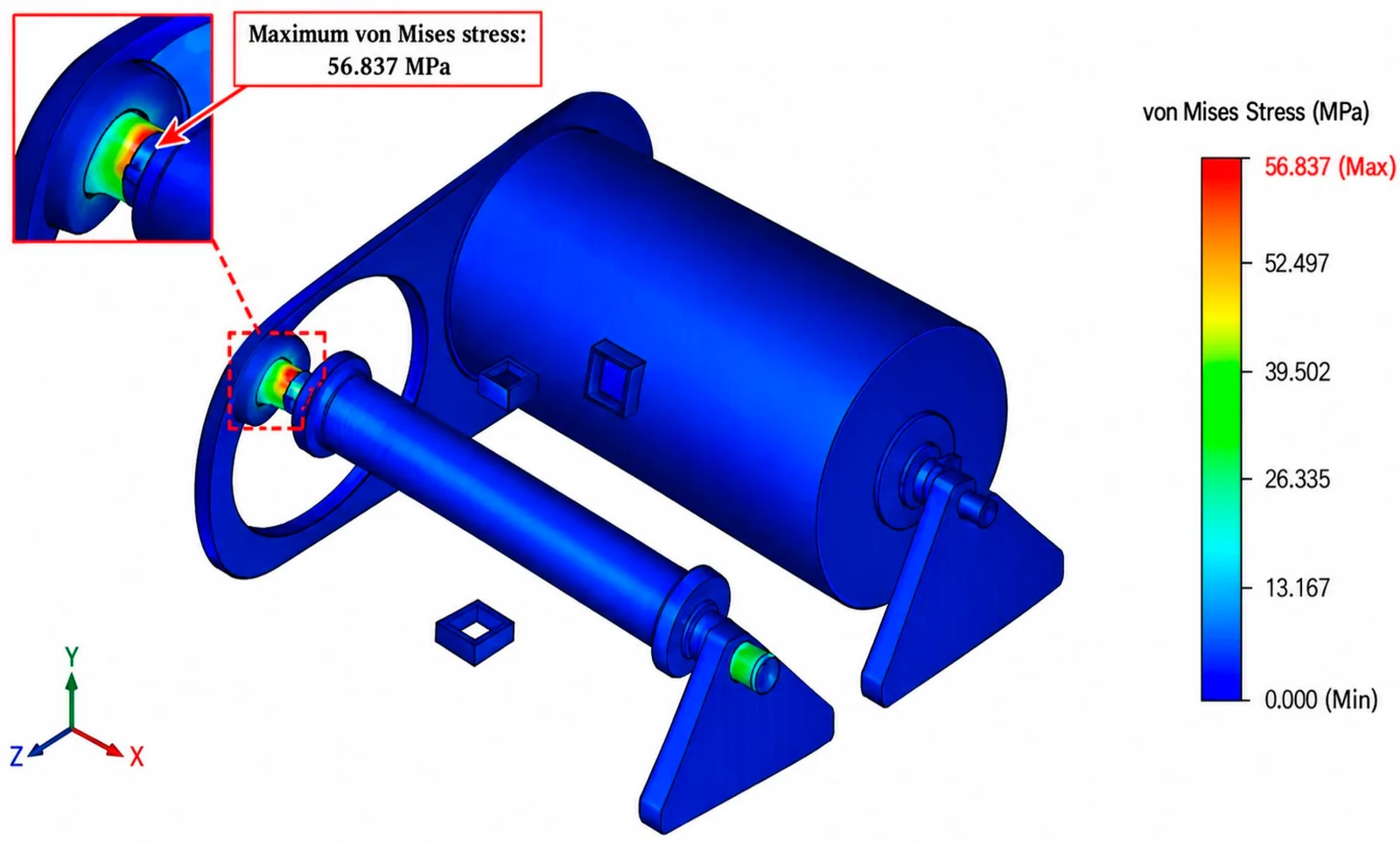
Structural response of the shaft–chamber–support assembly under torque load.

**Table 1:** Summary of calculated loads.

Load Component	Mass	Inertia-Force Amplitude	Gravitational Force
Mineral feed	50 kg	4.63 kN	0.49 kN
One grinding body	7 kg	0.65 kN	0.069 kN
Four grinding bodies	28 kg	2.59 kN	0.275 kN
Total internal load	78 kg	7.22 kN	0.765 kN
Maximum combined load	—	Approximately 7.98 kN	—

**Table 2:** Material properties assigned to the load-bearing components in the Autodesk Fusion simulations.

Property	Assigned Value
Density	7850 kg/m^3^
Young’s modulus	210 GPa
Poisson’s ratio	0.30
Yield strength	207 MPa
Ultimate tensile strength	345 MPa

## Data Availability

The original contributions presented in this study are included in the article. Further inquiries can be directed to the corresponding author.

## References

[1] Sterling D., Breitung-Faes S., Kwade A. (2023). Experimental Evaluation of the Energy Transfer within Wet-Operated Stirred Media Mills. Powder Technol..

[2] Napier-Munn T. (2015). Is Progress in Energy-Efficient Comminution Doomed?. Miner. Eng..

[3] Wills B.A., Finch J. (2015). Wills’ Mineral Processing Technology: An Introduction to the Practical Aspects of Ore Treatment and Mineral Recovery.

[4] Ratna Kumar P.S.S., St. John Alexis P., Yaragalla S., Mishra R., Thomas S., Kalarikkal N. (2019). Chapter Four. Synthesized Carbon Nanotubes and Their Applications. Carbon-Based Nanofillers and Their Rubber Nanocomposites.

[5] Fuerstenau D.W. (1995). Grinding Aids. KONA Powder Part. J..

[6] Jeswiet J., Szekeres A. (2016). Energy Consumption in Mining Comminution. Procedia CIRP.

[7] DisserCat. https://www.dissercat.com.

[8] Revnivtsev V.I., Kapralov E.P., Kostin I.M. (1985). Improvement of Crushing, Grinding, Screening and Classification Processes for Ores and Beneficiation Products.

[9] Nenarokomova Y.F. (1975). Improvement and Development of Ore Preparation Processes for Beneficiation. Proceedings of Mekhanobr.

[10] Lesin A.D., Rozhentsov I.V. (1989). Crushing and Grinding Equipment and Disintegration Technology. Interdepartmental Collection of Scientific Papers, Mekhanobr.

[11] Askarov E.S., Arinova D.B., Zhankeldi A.Z., Ilyasova A.K. (2017). Centrifugal gyration mill for grinding mineral raw materials. Bull. KazATK—Almaty.

[12] Askarov E.S. (2021). How to Learn to Invent: A Guide for a Novice Inventor.

[13] Mashekov S.A., Nugman E.Z., Mashekova A.S., Bekbosynova B.A., Tussupkaliyeva E.A., Absadykov B.N. (2020). Investigation of power and force parameters of pressing of precisions at the continuous pressing mill of new construction. News Natl. Acad. Sci. Repub. Kazakhstan Ser. Geol. Tech. Sci..

[14] Kumar A., Sahu R., Tripathi S.K. (2023). Energy—Efficient advanced ultrafine particle crushing using stirrer mills—An overview. Energy.

[15] Naeimi M., Behrad Vakylabad A., Hosseini S.M., Hassanzadeh A., Rahmanian A., Asgari K., Duan C., Nazari S. (2026). A Critical Review of Innovations in Stirred Mill Technology for Enhancing Energy Efficiency. Miner. Process. Extr. Metall. Rev..

[16] Elbendari A.M., Ibragim S.S. (2025). Optimization of key parameters to increase the energy efficiency of grinding and modeling of particle size distribution in a ball mill with stirring. Sci. Rep..

[17] Hosten S., Fidan B. (2012). Industrial comparative study of cement clinker crushing systems in terms of specific energy consumption and cement properties. Powder Technol..

[18] Tuil D., Belaadi S., Francis K. (2006). Energy efficiency of Cement finishing Grinding in a dry-acting periodic ball mill. Cem. Concr. Res..

[19] Andreev S.E., Perov V.A., Zverevich V.V. (1980). Crushing, Grinding and Screening of Minerals: A Textbook for Universities.

[20] Tumashev A.S., Avvakumov E.G. (2010). Engineering and Construction Group: Patent of the Republic of Belarus No. 2381070. IPC B02C.

[21] Semikopenko I.A., Voronov V.P., Belyaev D.A. (2024). Centrifugal Counterflow Mill with Classification of Material in the Loading Zone. Cem. Its Appl..

[22] Avvakumov E.G. (1986). Mechanical Methods of Activation of Chemical Processes.

[23] Khatir A., Capozucca R., Khatir S., Magagnini E., Le Thanh C., Riahi M.K. (2025). Advancements and emerging trends in integrating machine learning and deep learning for SHM in mechanical and civil engineering: A comprehensive review. J. Braz. Soc. Mech. Sci. Eng..

[24] Khatir A., Capozucca R., Khatir S., Magagnini E., Cuong-Le T. (2025). Enhancing damage detection using Reptile Search Algorithm-optimized neural network and Frequency Response Function. J. Vib. Eng. Technol..

[25] Khatir A., Oulad Brahim A., Capozucca R., Khatir S., Noori M., Sarhosis V., Bhowmik B., Cuong-Le T. (2026). Structural recovery and damage prediction in GFRP-BFRP patched plates using hybrid SSA-SCA-XGBoost model and vibration data. Compos. Struct..

[26] Bond F.S. (1952). The third theory of grinding. Trans. AIME.

[27] Saramak D., Leśniak K. (2024). Impact of HPGR operational pressing force and material moisture on energy consumption and crushing product fineness in high-pressure grinding processes. Energy.

[28] Yang F., Li R., Wang X., Cheng B., Gu R. (2024). Optimization of working parameters of high-pressure roller mill based on entropy weight method and response surface method. Sci. Rep..

[29] Shen L., Jiang X., Liu X., Liu H., Song S., Han Q., He X. (2024). Research on the Grinding Process of Superhard Particles in the Fluidized Bed Opposed Jet Mill Based on the CFD-DEM Methodology. Minerals.

[30] Huang S., Yin X., Wang H., Li H., Wang Z., Chen H. (2025). Improving the energy conversion efficiency between airflow and particles in fluidized bed opposed jet mill via a composite nozzle: Experiments and simulations. Chem. Eng. J..

[31] Buddhiraju V.S., Maheshwari S., Rai B., Moys M., Runkana V. (2024). Modeling and Simulation of Ultrafine Grinding of Alumina in a Planetary Ball Mill. Trans. Indian Inst. Met..

[32] Cabello R., Han J., Popescu A.E.P., Bonet-Ruiz J., Batlle G.C., Barreneche C., Dosta S. (2024). Discrete Element Method Optimization Simulation of Planetary Ball Mills Operating Conditions. Chem. Eng. Trans..

[33] Kursunoglu S. (2025). A Review on the Recovery of Critical Metals from Mine and Mineral Processing Tailings: Recent Advances. J. Sustain. Metall..

[34] Afanasyev A.I., Zubov V.V., Simisinov D.I., Chirkova A.A. (2025). Izmelchenie gornoi porody v tsentrobezhnykh melnitsakh [Grinding of rock in centrifugal mills]. Min. Informational Anal. Bull..

[35] Bogdanov V.S., Antsiferov S.I., Bogdanov D.V., Sychev E.A. (2022). Sostoyanie i napravleniya razvitiya tekhniki i tekhnologii izmelcheniya [State and development directions of grinding equipment and technology]. Bull. Belgorod State Technol. Univ. Named After. V. G. Shukhov.

[36] Absadykov B., Sanjin T., Kristina M., Željko V. (2024). Efficiency-based Methodology for the Selection of Electric Motors for Mini-tractor Propulsion. FME Trans..

[37] Dhakate M.M., Venkatraman A., Khakhar D.V. (2023). Breakage of a Single Particle in an Air Jet Mill. ACS Eng. Au.

[38] Dmitrak Y., Kravtsov A., Adamova L., Kutuev R., Tsyganov A. (2025). Nauchnyj analiz matematicheskih modelej dvizhenija meljushhej zagruzki v pomolnyh kamerah melnic razlichnyh tipov [Scientific analysis of mathematical models of charge motion in grinding chambers of various-type mills]. Min. Informational Anal. Bull..

[39] Fedarovich E.G., Levdansky A.E., Kovaleva A.A., Nurmukhamedov K.S. (2024). Ispolzovanie matematicheskikh modelei turbulentnosti dlya opredeleniya gazodinamicheskikh kharakteristik udarno-tsentrobezhnoi melnitsy [Use of turbulence mathematical models to determine gas-dynamic characteristics of an impact centrifugal mill]. Proc. BSTU Ser. 2. Chem. Eng. Biotechnol. Geoecology.

[40] Garkavi M.S., Kutlubaev I.M., Khozei A.B. (2016). Issledovanie dvizheniya materiala v kamere drobleniya tsentrobezhnoi drobilki i sovershenstvovanie konstruktsii uskoritelya [Study of material movement in the crushing chamber of a centrifugal crusher and improvement of the accelerator design]. Bull. Belgorod State Technol. Univ. Named After. V. G. Shukhov.

[41] Grebenchuk P.S., Levdansky E.I. (2013). Opisanie dvizheniya chastits materiala v rabochikh zonakh udarno-tsentrobezhnoi melnitsy [Description of particle motion in the working zones of an impact centrifugal mill]. Mech. Eng. Mag..

[42] Guryanov G., Doudkin M., Kim A., Vavilov A., Vasilyeva O. (2025). Improving the efficiency of fine grinding of materials in a new inverted impact grinder with enhanced energy-technological performance. Adv. Mech. Eng..

[43] Khetagurov V.N., Kamenetskii E.S., Gegelashvili M.V., Pliev V.A. (2018). Ispolzovanie tsentrobezhnykh melnits vertikalnogo tipa dlya razmola kuskovoi chasti flyusovykh materialov pri prigotovlenii aglomeratsionnoi shikhty [Use of vertical centrifugal mills for grinding lump flux materials in agglomeration charge preparation]. Bull. High. Educ. Inst. North Cauc. Reg..

[44] Levdanskiy A., Fedorovich E., Kovaleva A., Golubev V., Korganbayev B., Sarsenbekuly D., Zhumadullayev D. (2020). Integrated Study of the Efficiency of Grinding Material in an Impact-Centrifugal Mill. News Natl. Acad. Sci. Repub. Kazakhstan Ser. Geol. Tech. Sci..

